# Cache Domains That are Homologous to, but Different from PAS Domains Comprise the Largest Superfamily of Extracellular Sensors in Prokaryotes

**DOI:** 10.1371/journal.pcbi.1004862

**Published:** 2016-04-06

**Authors:** Amit A. Upadhyay, Aaron D. Fleetwood, Ogun Adebali, Robert D. Finn, Igor B. Zhulin

**Affiliations:** 1 Genome Science and Technology Graduate Program, University of Tennessee–Oak Ridge National Laboratory, Knoxville, Tennessee, United States of America; 2 Department of Microbiology, University of Tennessee, Knoxville, Tennessee, United States of America; 3 Computer Science and Mathematics Division, Oak Ridge National Laboratory, Oak Ridge, Tennessee, United States of America; 4 European Molecular Biology Laboratory, European Bioinformatics Institute, Wellcome Trust Genome Campus, Hinxton, Cambridge, United Kingdom; Icahn School of Medicine at Mount Sinai, UNITED STATES

## Abstract

Cellular receptors usually contain a designated sensory domain that recognizes the signal. Per/Arnt/Sim (PAS) domains are ubiquitous sensors in thousands of species ranging from bacteria to humans. Although PAS domains were described as intracellular sensors, recent structural studies revealed PAS-like domains in extracytoplasmic regions in several transmembrane receptors. However, these structurally defined extracellular PAS-like domains do not match sequence-derived PAS domain models, and thus their distribution across the genomic landscape remains largely unknown. Here we show that structurally defined extracellular PAS-like domains belong to the Cache superfamily, which is homologous to, but distinct from the PAS superfamily. Our newly built computational models enabled identification of Cache domains in tens of thousands of signal transduction proteins including those from important pathogens and model organisms. Furthermore, we show that Cache domains comprise the dominant mode of extracellular sensing in prokaryotes.

## Author Summary

Cell-surface receptors control multiple cellular functions and are attractive targets for drug design. These receptors often have dedicated extracellular domains that bind signaling molecules, such as hormones and nutrients. Computational identification of these ligand-binding domains in genomic sequences is a pre-requisite for their further experimental characterization. Using available three-dimensional structures of several bacterial cell-surface receptors, we built computational models that enabled identification of the Cache domain, as the most common extracellular sensor module in prokaryotes, including many important pathogens. We also demonstrated that the Cache domain is homologous to, but sufficiently different from the most common intracellular sensor module, the PAS domain. These findings provide a unified view on molecular principles of signal recognition by extra- and intracellular receptors.

## Introduction

**Fig 1:**
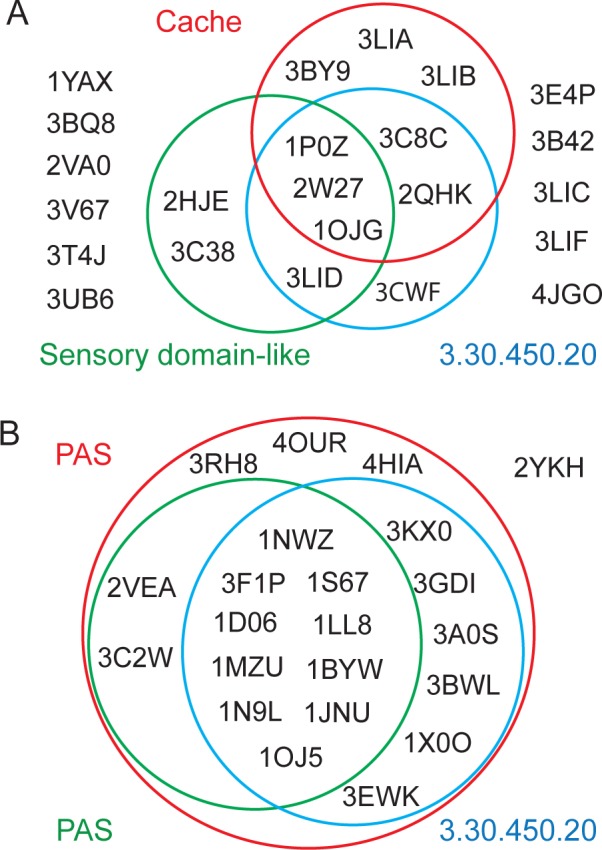
Superfamily assignment of PAS domains in sequence and structure classification databases. (A) Extracellular PAS-like domains; (B) intracellular PAS domains. Assignments of PDB structures by Pfam [26] (red), SCOP [27] (green) and CATH [28] (blue) are shown as Venn diagrams to scale.

Signal transduction is a universal feature of all living cells. It is initiated by specialized receptors that detect various extracellular and/or intracellular signals, such as nutrients, and transmit information to regulators of different cellular functions [1, 2]. Receptors are usually comprised of several domains and one or more of them are designated sensors that physically interact with the signal. There is a great diversity in the sensory domain repertoire, but a few of these domains appear to be dominant. The most abundant sensory module that is found in tens of thousands of signal transduction proteins throughout the Tree of Life is the Per/Arnt/Sim (PAS) domain [3, 4]. PAS domains are related to another large group of dedicated sensors–cGMP phosphodiesterase/adenylyl cyclase/FhlA (GAF) domains [5, 6]: both superfamilies belong to the profilin-like fold [6, 7] and are found in similar types of signal transduction proteins in eukaryotes and prokaryotes. PAS and GAF are amongst the largest superfamilies of small molecule-binding domains in general, and the largest among those solely dedicated to signal transduction [8]. Originally, PAS domains were discovered as exclusively intracellular sensors [9, 10]; however more recent studies have identified several extracytoplasmic PAS domains. Members of this group include quorum- [11], dicarboxylate- [12, 13] and osmo-sensing [14] receptor kinases, and chemotaxis receptors [15, 16] from bacteria as well as the *Arabidopsis thaliana* cytokinin receptor [17] among others. As commonly accepted in structure-based approaches, these domains were termed PAS (or PAS-like) based on expert’s visual inspection of three-dimensional structures. Surprisingly, none of these structurally defined domains matched any sequence-derived PAS domain models. Furthermore, novel structural elements previously unseen in PAS domains have been noticed in some of these structures and a new name, PDC (acronym of three founding members, PhoQ, DcuS and CitA), has been suggested for these extracellular domains [18]. On the other hand, several unappreciable, but independent observations pointed toward a possible link between extracellular PAS-like structures and yet another sensory domain superfamily, Cache [19]. Cache was originally described as a ligand-binding domain common to bacterial chemoreceptors [20] and animal voltage-dependent calcium channel subunits [21] that are targets for antineuropathic drugs [22]. First, the authors of the original Cache publication suggested that three predicted strands in the Cache domain might form a sheet analogous to that present in the core of the PAS domains structure; they also suggested a circular permutation of the Cache domain in extracellular regions of DcuS and CitA [19], proteins that later became the founding members of the proposed PDC domain [18]. Second, in their structural classification of PAS domains, Henry and Crosson [4] noted that a few sequences corresponding to structures included in their analysis were annotated as Cache in domain databases. Third, Zhang and Hendrickson reported that a conserved domain search detected the presence of a single Cache domain in their two related structures of the double PDC domain, namely 3LIA and 3LIB (PDB identifiers), but not in the other three closely related structures of this domain, 3LIC, 3LID and 3LIF [23]. Nevertheless, these potential relationships with Cache have never been explored further and extracellular PAS-like domains are being referred to as PAS [4], PAS-like [14], PDC [18], PDC-like [24], and PDC/PAS [25] (S1 Table). Furthermore, there is no agreement between sequence- and structure-based classifications of these domains and associated structures provided by leading databases (Fig 1, S2 and S3 Tables).

The fundamental problem beyond classification issues and semantics is that other than a handful of examples with solved 3D structure, receptors containing these domains cannot be identified by tools implemented in major biological databases, such as the NCBI Conserved Domain database [29], Pfam [26], SMART [30], etc. This, in turn, is a barrier for practical applications, such as a proposed use of bacterial receptors as drug targets [31]. On the other hand, Dunin-Horkawicz and Lupas [32] were able to detect many extracellular PAS-like domains in genomic datasets by using a sensitive profile-profile search tool HHpred [33] and PDB derived profiles, thus laying a foundation for further exploration of these complex sequence-structure relationships.

Here we show that extracellular PAS (PDC)-like domains belong not to PAS, but to the Cache superfamily. By building new Cache domain models utilizing structural information, we implicated more than 50,000 signaling proteins from all three domains of life as new members of this superfamily thus more than doubling the space of its current computational coverage. We also provide evidence that while being a distinct superfamily, Cache is homologous to the PAS superfamily and propose that the Cache domain emerged in bacteria from a simpler intracellular PAS ancestor as a benefit of extracellular sensing. Finally, we show that Cache domains are the dominant mode of extracellular sensing in prokaryotes.

## Results

### “Extracellular PAS” Is Cache

To illustrate the level of ambiguity in classification of extracellular PAS/PDC-like domains (S2 Table) we compared it to that of diverse intracellular PAS domains from bacteria, archaea and eukarya (S3 Table). The results show a nearly perfect classification coverage and agreement between sequence- and structure-based definitions for the latter and a state of disarray for the former (Fig 1). We subjected protein sequences of all twenty-one single and double extracellular PAS-like domains [4] with known 3D structure to similarity searches against the Pfam database (v.27.0) using sequence-to-profile search tool, hmmscan [34] and a more sensitive, profile-to-profile search tool HHpred [33]. None of the sequences had any PAS domain models as the best hit in any type of search. For fourteen of them (including both single and double domains), best hits were to domain models from the Cache superfamily, whereas for the remaining seven structures, best hits are not assigned to any domain superfamily (S4 Table).

**Fig 2:**
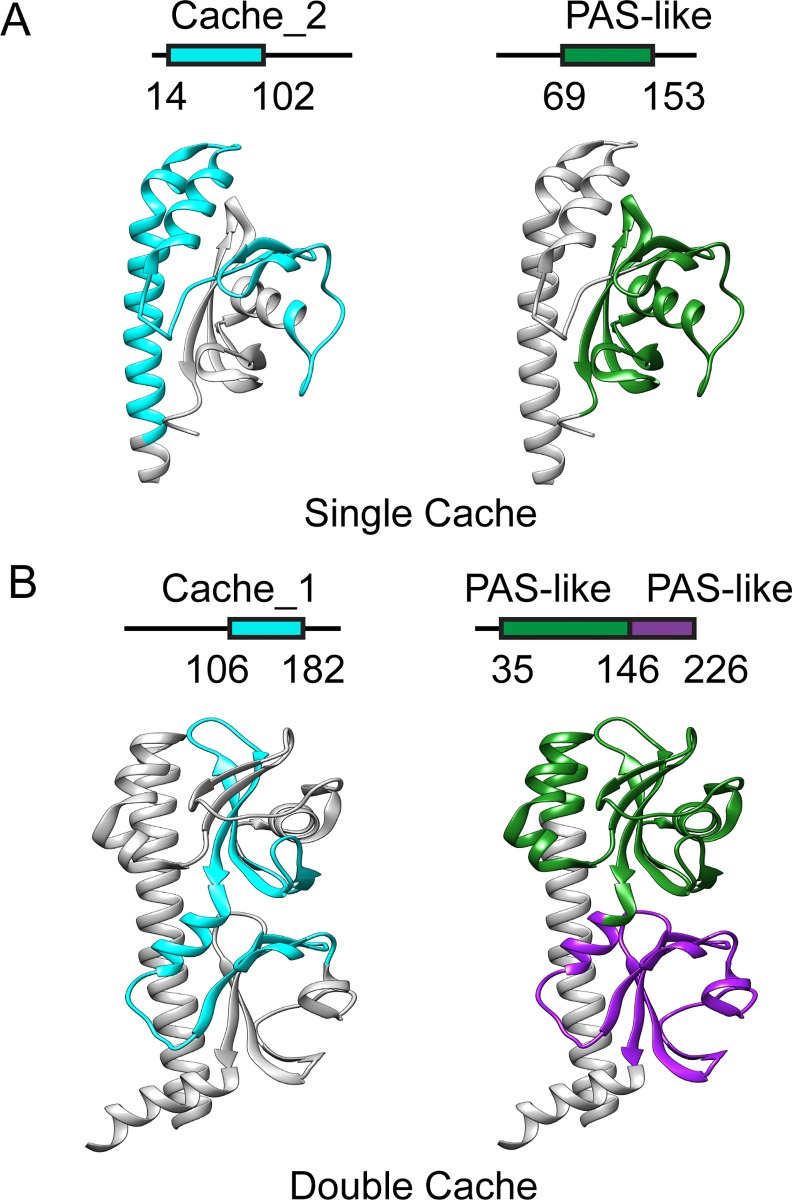
Comparison of sequence- and structure-based definitions for extracellular PAS-like domains. (A) *Vibrio parahaemolyticus* chemoreceptor (PDB: 2QHK); (B) *Vibrio cholerae* chemoreceptor (PDB: 3C8C). Domains are visualized on sequences with corresponding amino acid positions (top) and structures (bottom). Cache (cyan) domains are defined by Pfam; PAS domains (green and magenta) were defined by visual inspection of corresponding structures.

Mapping regions matched to Cache domains onto corresponding structures revealed the nature of ambiguity between sequence- and structure-based domain definitions. Single domain structures showed better agreement with sequence-based domain models (S1 Fig), although some of them still had substantial discrepancies. For example, the full-length Cache_2 model does not include the last three β-strands of the PAS-like domain (Fig 2A). Dual domain structures showed major disagreements with sequence-based domain models. The Cache_1 model captures the last three strands from the membrane distal PAS-like domain, the first two strands of the membrane proximal domain, and the connecting elements between the two domains (Fig 2B). Some of the most conserved structural elements, such as the long N-terminal helix captured in the Cache_2 model and connecting elements between two globular domains captured in the Cache_1 model, are never seen in proteins that belong to the PAS domain superfamily, which led to a suggestion that these domains are different from PAS [23]. We also confirmed that the long N-terminal helix in some of the double domain structures (Fig 2B) matches a Pfam model MCP_N (S4 Table).

### New Cache Domain Models

Cache domains were represented in Pfam 27.0 as a clan (Pfam definition of a superfamily) comprised of six families: Cache_1, Cache_2, Cache_3, YkuI_C, DUF4153 and DUF4173. We used newly uncovered relationships between structure and sequence characteristics to construct new Cache domain models. Three key facts about Cache domains were taken into account. First, structural studies revealed that both single and double Cache domains occupy the entire extracellular region between two transmembrane helices [11–17, 23]. Second, Cache domains have been identified exclusively in proteins that contain output signaling domains. Third, the vast majority of Cache domains are found in prokaryotes. Consequently, in order to identify potential Cache domains, we retrieved a non-redundant set of prokaryotic sequences that contained at least one output signaling domain and a predicted extracellular region flanked by two transmembrane helices (see Methods for details). The final set of predicted extracellular regions (non-redundant at 90% identity) was used in the hidden Markov model (HMM) construction. Models were built in three stages using sequence-to-sequence and HMM-to-HMM comparisons (see Methods for details). We constructed eight new Cache models (four double Cache models–dCache_1, dCache_2, dCache_3, Cache_3-Cache_2 and four single Cache models–sCache_2, sCache_3_1, sCache_3_2 and sCache_3_3) to replace the three models (Cache_1, Cache_2, and Cache_3) from Pfam 27.0 (Table 1). The alignments for the eight new models are shown in S1 Data. The fourth Pfam model from the Cache clan, YkuI_C, was found to adequately capture the domain structure and to perform well (S1B Fig). Two other members of the clan, DUF4153 and DUF4173 were found to be unrelated to Cache based on both sequence similarity and secondary structure prediction. Consequently, these models will be removed from the clan.

**Table 1:** Newly defined Cache superfamily.

Family	Total	HK	MCP	GCD	AC	SP	STK	IC	TF	PDB
					GC					
**Double domains**										
dCache_1	15569	4958	4908	2880	265	204	25	467	300	3C8C, 2ZBB, 3BY9, 3E4P, 3LIA, 3LIB, 3LIC, 3LID, 3LIF, 4JGO
dCache_2	299	71	92	89	-	21	-	-	1	-
dCache_3	883	327	236	248	4	8	1	-	-	-
Cache_3-Cache_2	407	17	330	10	-	-	-	-	-	-
CHASE	1214	607	-	549	9	3	5	-	-	3T4J
LuxQ-periplasm	115	112	-	1	-	-	-	-	-	2HJE, 3C38
**Single domains**										
sCache_2	2243	356	1534	29	-	2	-	-	-	2QHK,3UB6,4K08
sCache_3_1	2854	2799	3	15	-	1	2	-	3	3CWF
sCache_3_2	2499	2189	64	40	-	60	2	-	3	1P0Z, 3BY8
sCache_3_3	276	14	201	15	-	-	-	-	14	-
YkuI_C	277	-	-	178	-	-	-	-	-	2W27
CHASE4	529	79	7	387	3	-	-	-	-	-
Stimulus_sens_1	203	202	-	-	-	-	-	-	-	-
DUF2222	713	705	-	1	-	-	-	-	-	-
SMP_2	788	-	-	-	-	-	-	-	-	-
Diacid_rec	1274	-	30	3	-	-	-	-	1192	-
2CSK_N	966	952	-	-	-	-	-	-	-	2KSE
PhoQ_sensor	556	551	-	-	-	-	-	-	-	3BQ8, 1YAX

Number of sequences in UniProt 2012_06 release are shown. Abbreviations: MCP, methyl-accepting chemotaxis proteins (MCPsignal); HK, histidine kinases (HATPase_c, HATPase_c_2, HATPase_c_3, HATPase_c_5, HisKA, HisKA_2, HisKA_3, HWE_HK); GCD, c-di-GMP-cyclases and diesterases (GGDEF, EAL, HD); SP, serine phosphatases (SpoIIE, PP2C, PP2C_2); AC/GC, adenylate- and guanylate cyclases (guanylate_cyc): STK, serine/threonine kinases (Pkinase); TF, transcription factors (HTH clan, LytTR); IC, ion channels (VWA_N, VGCC_alpha2).

The new models revealed complex relationships between single and double Cache domains. HMM-HMM comparison (see Methods) showed that the membrane distal subdomain of dCache_1 was more similar to sCache_3, whereas the membrane proximal subdomain was more similar to sCache_2 (S2 Fig). On the other hand, dCache_2 and dCache_3 domains appear to be a result of sCache_2 and sCache_3 duplication, respectively. Finally, the Cache_3-Cache_2 domain likely originated as a fusion of sCache_3 and sCache_2 domains.

The new models demonstrated dramatically improved sensitivity by identifying more than 50,000 Cache domains in the NCBI non-redundant database that escaped detection by Pfam 27.0 models (S2 and S3 Data, S5 Table). HMM-HMM comparisons of newly identified Cache domains were carried out against the HHpred PDB70 profile database. 91% of the newly identified Cache domains were found to hit the PDB profile generated from available structures of the extracellular “PAS-like” domains (S4 Data). The results further support that the newly generated models correctly identify Cache domains.

A small number of newly identified Cache domains (~4%) overlapped with other non-Cache Pfam domains, such as MCP_N, TarH, VGCC_alpha2 and few others (S5 Data). As already discussed earlier, we consider MCP_N as a part of the Cache domain as it defines a subset of conserved Cache structural elements. Overlap with TarH is caused by inclusion of several Cache-domain containing sequences in the seed alignment for a model depicting an all alpha-helical TarH domain [35]. VGCC_alpha2 is usually present C-terminal to the Cache domain in Calcium channel subunits and in fact is a C-terminal part of the Cache domain missing from a Pfam 27.0 seed alignment. After correcting for these artifacts, the overlap of newly defined Cache domains with unrelated Pfam domains is about 0.15%.

**Fig 3:**
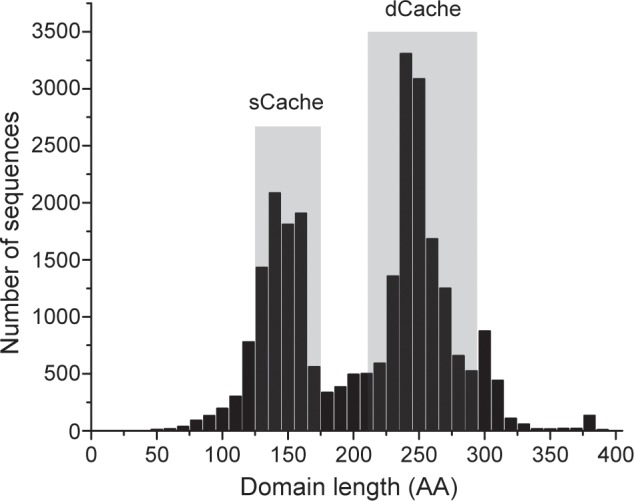
Length distribution of Cache domains identified using the new domain models. Results for searches of the Pfam 27.0 associated UniProt database (June 2012 release) using the newly built single and double Cache models and the unchanged YkuI_C model are shown. Shaded areas show the upper and lower boundaries of known single and double Cache domain structures. Outliers represent partial protein sequences as well as partial matches to models (very short sequences) and sequences with large insertions within the Cache domain (very long sequences). See S2 Data for details.

New models also showed a significantly improved average coverage (Fig 3, S6 Table). The average length of single and double Cache domains of known 3D structures is 140 and 271 amino acid residues, respectively, matching well previously observed bimodal distribution of extracellular ligand-binding regions in chemoreceptors [36]. Occasionally, single Cache domain models match to extracellular regions that are significantly larger than the average length of single Cache domains (S3 Fig). Similarly, double Cache domain models occasionally match to extracellular regions with a size of a single Cache domain. This is likely due to the complex modular nature of these domains (S2 Fig). We used sequences with known 3D structures as controls to visualize the increased specificity and coverage of the newly built Cache models (S7 Table). All new models, further refined according to Pfam standard protocols, are now available in the Pfam 29.0 release.

### New Members of the Cache Superfamily and Its Relationship to PAS and GAF

When carrying out sensitive profile-to-profile searches initiated with the sequences of extracellular “PAS-like” structures, we noticed statistically significant (although never the best) hits with profiles corresponding to several Pfam domains other than members of the current Cache clan. We explored this indication of potential remote homology further by consistently analyzing all statistically significant HHpred matches for all nineteen structures. The results show that statistically significant hits belong either to the PAS and GAF superfamilies or to small families that have not been assigned to any domain superfamily, for example LuxQ-periplasm, CHASE, Diacid_rec, etc (S6 Data, spreadsheet 1). Nearly the same repertoire of small families and members of PAS and GAF superfamilies were statistically significant hits in HHpred searches initiated with newly constructed Cache models (S6 Data, spreadsheet 2). Finally, we have performed a reverse search, where queries were models from small families as well as PAS and GAF superfamilies identified as statistically significant hits in the previous two types of searches (S6 Data, spreadsheet 3). These searches have identified nine additional current Pfam families that lacked any superfamily assignments. We now assign these families to the Cache superfamily (see Methods, Table 1, S6 Data, spreadsheet 4). The sequence logos for all the members of the new Cache superfamily are shown in S7 Data.

**Fig 4:**
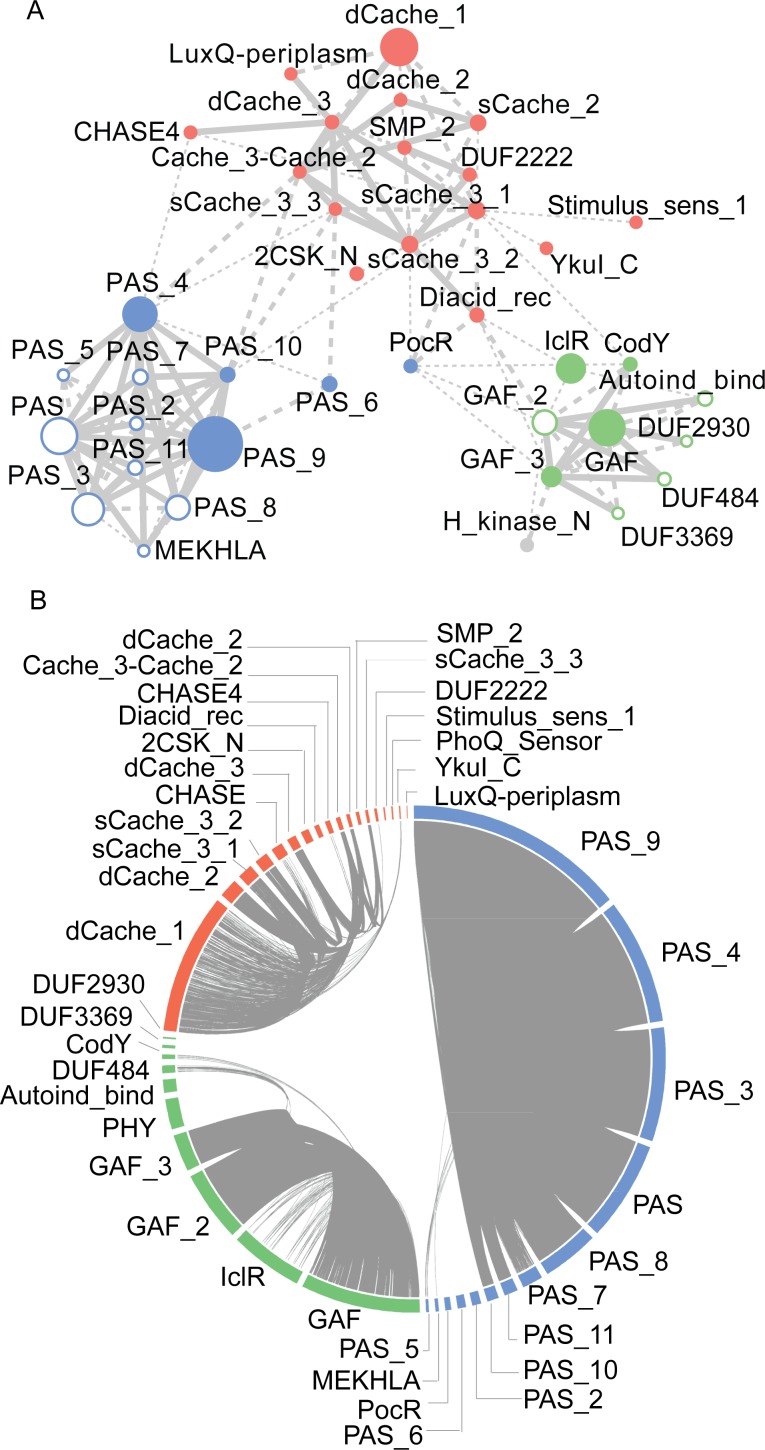
Relationship between Cache (red), PAS (blue) and GAF (green) superfamilies. (**A**) HMM-to-HMM comparisons. The nodes represent domain families. Links represent reciprocal hits in hhsearch. Hits with an E-value <1e-3 are shown as thick lines, those with E-value <1e-1 are shown as thin lines and dotted lines represent hits with >90 probability score. Filled circles represent PAS and GAF domain families that were identified in HHpred search using new Cache models. Families that were not identified in these searches are depicted by empty circles (**B**) Sequence-to-sequence comparisons. The outer circle represents domain families. Links between individual sequences represent reciprocal BLAST hits with an E-value threshold of 1e-8, the lowest E-value at which no links between superfamilies were found. However, the overall relationships shown here remain at less stringent E-values.

Relationships between all members of the Cache, PAS and GAF superfamilies at profile and sequence levels are shown in Fig 4. The clustered heat map (S4 Fig) generated using HHsearch Prob scores, shows four main clusters, one each for PAS, GAF and Cache superfamily and a fourth cluster comprising of several new Cache family members along with some smaller GAF and PAS families. While being closely related to PAS and GAF, members of the Cache superfamily are more related to each other, thus fully justifying a separate superfamily designation. Satisfactorily, homologous relationships between Cache, PAS, and GAF were also captured in a new database ECOD (Evolutionary Classification of Protein Domains) [37], which also included most of the related “orphan” families described above into the same superfamily.

A key unsolved biological problem in signal transduction is linking computationally derived models of sensory domains with their ligands. We have compiled a comprehensive literature survey, which showed that only a handful of Cache domains have known ligands (S8 Table). While it is unlikely that proposed models for individual Cache families capture the ligand-specific information (see Discussion), there seem to be at least some interesting trends. For example, the majority of known ligands for dCache_1 domains are amino acids, whereas many single Cache domains bind organic acids. Interestingly, no sugars were identified so far as ligands for Cache domains.

### Cache Domains Are Ubiquitous Extracellular Sensors

**Fig 5:**
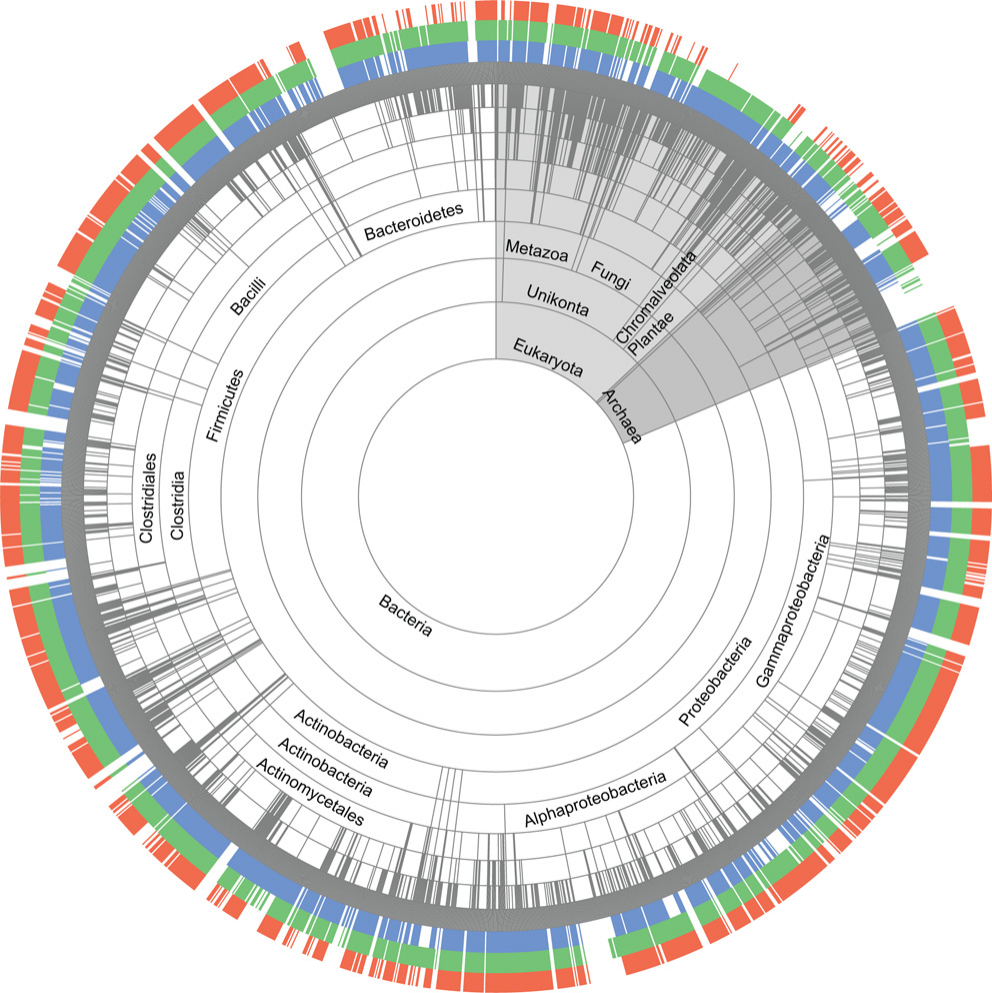
Phyletic distribution of PAS (blue), GAF (green) and Cache (red) domains. Flags at the outer three layers represent the domain presence in a corresponding genome. The tree was built using taxonomic ranks retrieved from NCBI.

By performing the hmmsearch against the Pfam 27.0 associated UniProt database using eighteen domain models from the newly defined Cache superfamily, we have identified 31,572 protein sequences containing these domains. Thus, the size of the Cache superfamily is comparable to that of PAS (88,093 sequences) and GAF (47,618 sequences) superfamilies. Overall phyletic distribution of Cache domains is also similar to that of PAS and GAF (Fig 5, S8 Data).

**Fig 6:**
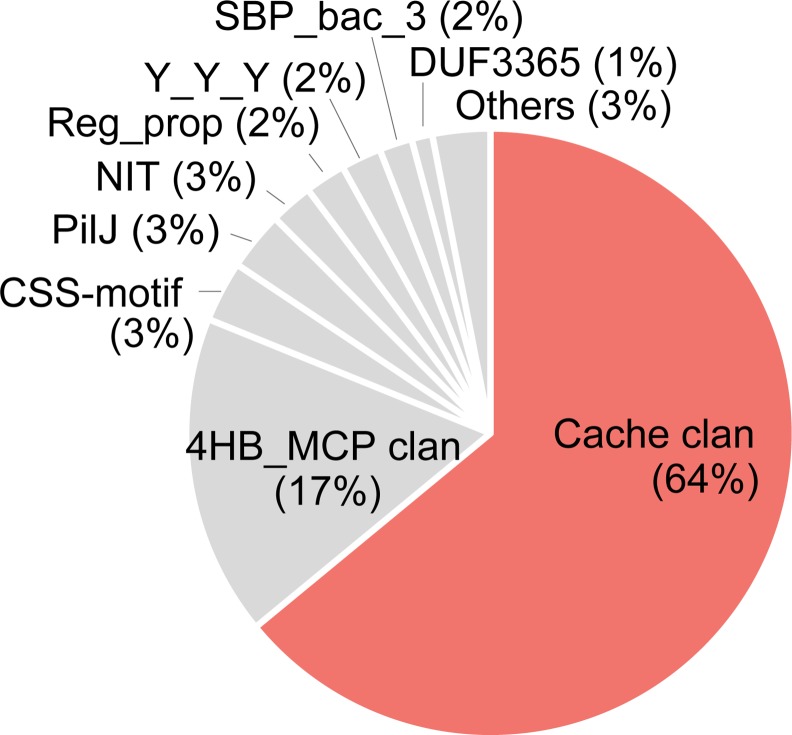
Relative abundance of known extracellular sensory domains in prokaryotes. Domain counts were obtained by running Pfamscan against a dataset of non-redundant prokaryotic extracellular sequences, which was also used for HMM construction (see Methods).

We have used the TMHMM2 tool to identify transmembrane regions in all 31,572 sequences with detectable Cache domains and determined that members of all Cache families are predicted to be principally extracellular, except for two small families, Diacid_rec and YkuI_C that are principally intracellular (S9 Table). Altogether, 78% of all Cache domains were confidently predicted to be extracellular. For comparison, 74% of all PAS domains were confidently predicted to be intracellular. Analysis of the domain architecture of all Cache domain-containing protein sequences revealed known output domains of signal transduction systems, except for the SMP_2 family members (Table 1). The SMP_2 domain is the closest relative of the DUF2222 domain (mutual best hits in HHpred searches) and both are found exclusively in proteobacteria. While DUF2222 is the sensory module of the BarA/GacS/VarA-type histidine kinases that are global regulators of pathogenicity in gamma-proteobacteria [38], SMP_2 appears to be a sensory module that was cut off from the rest of the protein. The likelihood of this scenario is further supported by the nearly identical phyletic distribution of both domains and the fact that SMP_2 proteins are also implicated in virulence in gamma-proteobacteria [39]. Apart from this neofunctionalization, all other Cache domains appear to serve as extracellular sensory modules for all major modes and brands of signal transduction proteins in prokaryotes, including sensor histidine kinases, cyclic di-GMP cyclases and diesterases, chemotaxis transducers, adenylate and guanylate cyclases, etc. Furthermore, Cache domains are dominant among known extracellular sensory domains in prokaryotes (Fig 6, S10 Table), significantly outnumbering the best studied such domain, a four-helix bundle [35, 40].

### Newly Identified Cache Domains

**Fig 7:**
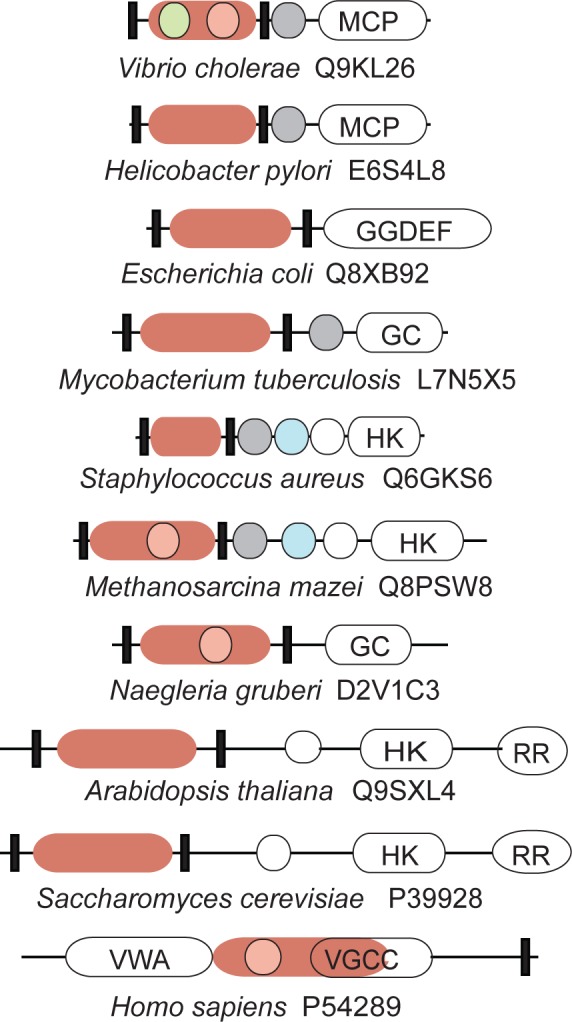
Examples of newly identified and better defined Cache domains in diverse signal transduction proteins from bacteria, archaea and eukaryotes. Domain architectures for representative sequences from model organisms are shown along with their UniProt accession numbers. Newly defined Cache domains are shown in red. Cache boundaries defined by the previous Pfam models are shown in pink (Cache) and green (MCP_N). HAMP domains are shown as grey circles, PAS domains as cyan circles, and HisKA domains as white circles. Other Pfam domains are abbreviated as follows: MCP, MCPsignal; GGDEF, GGDEF; GC, guanylate cyclase; HK, the histidine kinase HATPase_c domian; RR, response regulator; VWA, a combination of VWA_N and VWA domains; VGCC, VGCC_alpha2.

Among tens of thousands of newly identified Cache domains, many are present in signal transduction proteins from important human pathogens and model systems (Fig 7). For example, we have confidently detected the Cache domain in the extracellular region of the WalK sensor histidine kinase from low G+C Gram positive bacteria, which plays a critical role in regulating cell division and wall stress responses [41]. WalK is a novel target for antibacterial agents against multidrug-resistant bacteria, including methicillin-resistant *Staphylococcus aureus* [31, 42]. We identified the new double Cache domain in the YedQ diguanylate cyclase, which regulates cellulose biosynthesis and biofilm formation in *Escherichia coli* and *Salmonella enterica* [43, 44]. This domain was also identified in the Rv2435c adenylate cyclase in *Mycobacterium tuberculosis*, which is a part of the cAMP network involved in virulence [45]. Our new dCache_1 model has identified the double Cache domain in the extracellular region of the osmosensing histidine kinase Sln1 from *Saccharomyces cerevisiae*, which controls activity of the HOG1 pathway [46]. The region, which is now designated as the Cache domain, was shown to be essential for its sensory function [47].

### Evolutionary Scenario for Cache Origins

A meaningful phylogenetic tree of Cache domains cannot be produced due to extreme sequence variation between families. Consequently, evolutionary analysis of Cache is limited to less informative options. However, phyletic distribution, relative abundance and protein context all point towards a probability that Cache domain(s) evolved from simpler intracellular PAS-like ancestor(s). We have shown that Cache is homologous to PAS and GAF (Fig 4A), which is also independently supported by CATH [28] and ECOD [37] classification. PAS and GAF (that are homologous to each other) or their common ancestor originated in the last universal common ancestor [5, 8, 48]. Cache has all basic structural elements of PAS, but also contains novel structural elements that are not seen in PAS/GAF [23] including a long N-terminal helix previously mistaken for a separate domain (MCP_N). Thus, PAS and GAF are structurally simpler than Cache. Domains that are structurally simpler are expected to be more ancient and more abundant than their structurally more complex derivatives [49]. In bacteria, PAS, GAF, and Cache domains are nearly equally abundant, whereas in archaea and eukarya Cache is significantly less abundant suggesting that Cache has likely originated in the bacterial lineage after its separation from the archaeal/eukaryotic lineage. Incidences of Cache in archaea and eukaryotes appear to be due to horizontal gene transfer. For example, Cache domains in Metazoa are mostly limited to a single type of protein–a voltage-dependent calcium channel alpha-2-delta subunit [21] (S8 Data), whereas vertically inherited PAS and GAF domains are found in diverse signal transduction proteins [3, 50]. In plants and fungi, Cache is limited to histidine kinases (S8 Data) that are known to be horizontally transferred from bacteria [51, 52]. In *Naegleria*, a representative of Excavates, the Cache domain is found in a single protein, a bacterial-type adenylate cyclase (Fig 7). In a striking contrast, Cache domains in bacteria are found in all major types of signal transduction proteins (Table 1) similarly to PAS and GAF, and their phyletic distribution and abundance in bacteria are similar to that of PAS and GAF. Finally, the Cache-to-PAS ratio in archaea and eukaryotes is nearly five times smaller than that in bacteria (S8 Data). Taken together, these observations suggest that PAS and GAF predate Cache, which is consistent with the previous suggestion that intracellular sensing predates extracellular sensing [53].

## Discussion

Our findings show that experimentally solved three-dimensional structures of so-called “extracellular PAS domains” belong not to PAS, but to Cache superfamily. Our new sequence profile models for the Cache superfamily dramatically improve computational coverage and enable identification of Cache domains in tens of thousands of signal transduction proteins including those from human pathogens and model systems. Consequently, we demonstrated that Cache is the most abundant extracellular sensory domain in prokaryotes, which probably originated from a simpler intracellular PAS/GAF ancestor as a benefit of extracellular sensing. The key structural innovation in Cache domains, when compared to PAS and GAF, is the long N-terminal alpha helix (Fig 2), which is a direct extension of the first transmembrane helix. It appears that this simple innovation (along with a helical extension of the C-terminus to connect it to the second transmembrane helix) was sufficient to convert an intracellular sensor to an extracellular sensor. However, this also placed significant physical constraints on the ability of the sensor to transmit information. Intracellular PAS and GAF domains have multiple options for interacting with downstream signaling domains, including direct domain-to-domain binding. In contrast, the only option for an extracellular Cache to transmit signals is via its C-terminal transmembrane helix, similarly to the sensory four-helix bundle exemplified by the *E*. *coli* aspartate chemoreceptor [54]. It is highly likely that these physical constraints dictated some re-wiring of the PAS/GAF-like core in Cache domains resulting in evolutionary conservation of amino acid positions that are not under such constraints in cytoplasmic PAS and GAF domains. Although our new domain models and expansion of the Cache superfamily helped to newly identify tens of thousands of Cache domain-containing proteins in hundreds of species, the key biological question–what do these Cache domains sense–remains unanswered. At this time, only a handful of Cache domains have known ligands (S8 Table) and high sequence variation essentially prohibits the computational identification of function-specific positions for various Cache domains. This is a persistent problem in signal transduction. Changes in just two or three amino acid positions in the ligand-binding site can convert a serine sensor into an aspartate sensor [55] and in case of a covalently bound cofactor a single amino acid residue may define the receptor specificity [56]. On the other hand, certain trends connecting different Cache families to specific ligand classes can be observed. For example, the majority of known ligands for dCache_1 domains are amino acids, whereas organic acids comprise the major known class of ligands for single Cache domains (S8 Table). High-throughput screens, such as the one recently developed for microbial chemoreceptors [57], should lead to substantial expansion of the known ligand repertoire for Cache domains. Once various ligands are identified for different Cache domains, a computational analysis aiming at linking specific ligands (or ligand classes) to conserved sequence features may become productive. Finally, our results demonstrate that solving ambiguous sequence- and structure-based domain definitions can dramatically improve computational models and significantly accelerate computational coverage of the protein sequence space [58].

## Materials and Methods

### Data Sources and Bioinformatics Software

The central data source for all analyses was the local MySQL Pfam 27 [26] database based on Uniprot 2012_06 release. The database files for PfamScan were downloaded in December 2014. The Non-redundant database fasta file was retrieved from NCBI on April 2015. Uniref90 (April 2015) was used for running Psipred [59, 60]. The following software packages were used in this study: BLAST 2.2.28+ [61, 62], HHsuite-2.0.16 [33, 63, 64], CD-HIT 4.5.7 [65], Cytoscape 2.8.3 [66], BLAST2SimilarityGraph plugin for Cytoscape [67], Graph-0.96_01 (UnionFind) Perl library, MAFFT v7.154b [68], Jalview v2.7 [69], TMHMM 2.0c [70], Phobius v1.01 [71], DAS-TMfilter (December 2012) [72], HMMER 3.0 (March 2010) [34], PfamScan (October 2013) [26], MEGA 5.05 [73], Circos v0.64 [74] and Psipred v3.5. The multiple sequence alignments were built with MAFFT-LINSI using legacygappenalty option. Maximum likelihood trees were constructed to aid in the model building using MEGA with pairwise deletion and the JTT substitution. Domain predictions with PfamScan and hmmsearch were carried out at sequence E-value and domain E-value thresholds of 1E-3 for new Cache models and default thresholds for other Pfam models. Sequence logos were generated using the Skylign web-server [75].

### Hidden Markov Model Construction

A flow chart showing the model building approach is shown in S5 Fig. More than 1 million sequences containing at least one signal transduction output domain as defined in MiST2 database [76] were retrieved from a local copy of the Pfam database (S5 Fig). Eukaryotic sequences were discarded, because domain boundaries for Cache domains in eukaryotes are unclear. Predicted extracytoplasmic regions that were longer than 50 amino acids were scanned for Pfam domains and redundancy (at 90% identity) was removed resulting in 36,320 sequences. In the next step, a similarity network was built using the BLAST2similarityGraph Cytoscape plugin. Nodes were connected by edges if the blast alignment resulted in an E-value less than 1E-10 and a query coverage of >95% reciprocally. Each connected component was considered as a distinct cluster. At this threshold the known families of Cache–Cache_1, Cache_2, Cache_3 and YkuI_C were separated into distinct clusters. 38 clusters comprising of at least ten members and containing at least one Cache domain (7577 sequences in total) were further chosen for building models. Representative sequences were obtained using a custom script (S6 Fig) for each cluster and the sequences in each cluster were aligned using MAFFT-LINSi with the legacygappenalty option [77]. In case of the largest cluster, which was primarily comprised of sequences with the Cache_1 domain, the alignment was improved by dividing the cluster into smaller groups based on a maximum-likelihood tree generated using MEGA [78]. Individual groups were realigned using MAFFT-LINSi.

HMM models for each cluster were built using hhmake and all-against-all HMM-HMM comparison was carried out using HHsearch [64]. Based on the probability scores and coverage, the clusters were then merged using mafft-profile. Representatives of each cluster were chosen to construct HMMs using the hmmbuild utility in the HMMER3 package [34]. The sensitivity of the models was improved by incorporating remote homologs that were identified by a more sensitive HMM-HMM comparison using HHblits and HHsearch [63, 64].

### Representative Sequences

This algorithm outputs representative sequences for a given set of sequences based on all-against-all blast results (S6 Fig). Each query sequence is considered to be a representative of all hits that meet a certain threshold E-value and query coverage. The set of hits for a given query will be referred to as the represented set and the query sequence as the representative sequence. In order to reduce redundant computation, represented sets that were identical or subsets were discarded. The representative sequences were sorted based on the size of the represented set. The sequence with the largest represented set was first added to the list of representative sequences and the represented sequences were added to a new set, which we will refer to as the working set. Iteratively, a representative sequence was added to the list of representatives and the corresponding represented sequences are added to the working set. In each iteration, the representative sequence chosen was the one that results in the largest working set of represented sequences. Sequences were added to the list of representatives until all sequences that were provided as input have been included in the working set.

### HMM-HMM Comparison

The newly identified Cache sequences that were not detected with Pfam models were used to carry out HMM-HMM comparisons with HHpred PDB70 profile database (Sep 2015) in order to detect similarity to Cache domains with known structures. 638 sequences that were not in NCBI non redundant database (Feb 2016) were excluded. HHblits was first run to generate profiles for newly identified Cache sequences and HHsearch was then used to identify PDB hits for each sequence.

### New Members of the Cache Superfamily and Its Relationship to PAS and GAF

The sequences of extracellular PAS-like domains with available PDB structures were used as queries for HHpred search using default parameters against Pfam 27 database. Only hits with a probability score greater than 95 for at least one of the PDB queries were considered. The alignments used for creating the new Cache models were also used as queries for performing profile-profile comparisons using the HHpred web server against Pfam 27 database. All hits with a probability score greater than 70 were considered to be potentially homologous. To further explore the relationship between the families, we retrieved models for these hits along with new Cache models and the PAS and GAF clan. All-against-all HMM-HMM comparison was carried out using standalone hhsearch. A similarity network was created with the domain families as nodes and hits representing reciprocal hhsearch hits with (i) E-value less than 1E-3 (ii) E-value less than 1E-1 and (iii) probability score > 90. The E-value thresholds of 1E-3 and 1E-1 were used in accordance with the thresholds presently used in Pfam to define members of a clan (Pfam definition of a superfamily). In addition the threshold probability score of 90 was used to detect more remote relationships. The nodes in the network were manually rearranged after using unweighted Force-directed Layout. Families were assigned to the Cache clan when the E-value from HHpred was less than 1E-3 (LuxQ-periplasm, CHASE4, Diacid_rec and DUF2222) or when Cache was the closest superfamily (CHASE, Stimulus_sens_1 and 2CSK_N). SMP_2 and PhoQ_Sensor were included in Cache clan as they are mutual best hits with DUF2222 and 2CSK_N respectively.

A clustered heat map was also constructed using HHsearch Prob scores from HMM-HMM comparison. The Heatmap web server (http://www.hiv.lanl.gov/content/sequence/HEATMAP/heatmap.html) was used to carry out hierarchical clustering using threshold Prob score of >20, Euclidean distance method and Ward clustering.

We also performed sequence-sequence comparisons using all-against-all BLAST. The sequences for PAS clan, GAF clan and Cache clan comprising of new families were retrieved. For Cache clan, sequences that have overlapping domain prediction with other sensory Pfam domains were disregarded. 100% redundant sequences were removed using CD-HIT. The similarities between different domains were demonstrated using Circos tool [74].

### Phyletic Distribution of Cache, PAS and GAF Families

In order to show the phyletic distribution, only those organisms having more than 1000 proteins in Pfam 27.0 database were selected to exclude organisms with relatively incomplete genomes. The Sunburst was created by clustering the main level taxonomic ranks retrieved from NCBI Taxonomy database with the lowest rank used that of species. The domains were considered to be present if any strain of a given organism was found to contain a given domain. The Sunburst was generated using a custom script. PAS and GAF clans include all the families defined in Pfam 27.0. However, the Cache domains indicated comprise of those identified by the eight new models, YkuI_C as well as the other families (2CSK_N, CHASE, CHASE4, Diacid_rec, DUF2222, LuxQ-periplasm, PhoQ_Sensor, SMP_2 and Stimulus_sens_1) that were identified to be a part of the Cache clan in this study.

### Cache Dendrogram

The secondary structure prediction by Psipred was mapped on to the alignment for each model. Only the PAS-like regions comprising of five beta strands were extracted. HMM profiles were built for each alignment using hhmake tool in the HHsuite. All-against-all HMM-HMM comparison was performed using hhsearch. A distance matrix was generated using probability scores from hhsearch. The dendrogram showing similarity between single Cache domains and the membrane-distal and membrane proximal domains of double Cache was generated using the DendroUPGMA web server [79].

## Supporting Information

S1 Fig Comparison of sequence- and structure-based definitions for extracellular and intracellular single PAS-like domains. (A) Periplasmic domain of CitA from *Klebsiella pneumoniae* (PDB-1P0Z). Cache_3 domain is shown in cyan, (B) YkuI comprising of EAL and YkuI_C domains from *Bacillus subtilis* (PDB-2W27). The EAL domain is shown in gray and YkuI_C domain is shown in cyan. The Pfam based domain predictions for Cache_3 and YkuI_C map to distinct structural domains in contrast to Cache_1 and Cache_2 that map only to parts of structural domains (Fig 2).
(TIF)

S2 Fig Relationship between single Cache domains and the membrane distal and membrane proximal domains of double Cache. The PAS-like regions were extracted for each model based on secondary structure prediction and all-against-all HHsearch comparison was carried out. The dendrogram was generated by using the probability scores as similarity measure.
(TIF)

S3 Fig Coverage of extracellular regions by new Cache models. (A) dCache_1, (B) dCacche_2, (C) dCache_3, (D) Cache_3-Cache_2, (E) sCache_2, (F) sCache_3_1, (G) sCache_3_2, (H) sCache_3_3.Scatterplot showing relationship between the length of the extracellular regions and the percent query coverage of the extracellular regions by the new Cache domain models.
(TIF)

S4 Fig Clustered heat map of Cache, PAS and GAF superfamilies using HHsearch HMM-HMM comparison. The HHsearch Prob scores were used to generate the heatmap using a threshold Prob score of > = 20, Euclidean distance and Ward clustering using the Heatmap tool http://www.hiv.lanl.gov/content/sequence/HEATMAP/heatmap.html.
(TIF)

S5 Fig Flow chart of the HMM construction process. (TIF)

S6 Fig Algorithm for selecting representatives from a given set of sequences based on all-against-all BLAST results. (TIF)

S1 Table Timeline of PAS, Cache and PDC domain discoveries. (DOCX)

S2 Table Family (domain) and superfamily assignments for extracellular PAS-like domains. (DOCX)

S3 Table Family (domain) and superfamily assignments for intracellular PAS domains. (DOCX)

S4 Table Best Pfam database matches for extracellular PAS-like domains in sequence-profile and profile-profile searches. (DOCX)

S5 Table Number of Cache domains predicted by Pfam 27 Cache models and new models against Pfam 27 associated UniProt database (June 2012 release) and NCBI non-redundant (NR) database (April 2015 release). (DOCX)

S6 Table Query coverage of extracellular regions by new Cache domain models. The query coverage was determined by dividing the length of predicted Cache domain over the length of the extracellular region. The frequency distribution table shows the percentage of Cache domains for different query coverage intervals (bin = 10).
(DOCX)

S7 Table Computational coverage of Cache domains in proteins with known 3D structure. The query coverage was determined by dividing the length of predicted Cache domain over the length of the extracellular region for the PDB sequence.
(DOCX)

S8 Table Known ligands for members of the Cache superfamily. (DOCX)

S9 Table Cellular localization prediction for members of the Cache superfamily using TMHMM. (DOCX)

S10 Table Abundance of the two largest clans among known prokaryotic extracellular sensory domains. Domain models were searched against non-redundant prokaryotic extracellular sequences
(DOCX)

S1 Data Seed alignments for new Cache models. (PDF)

S2 Data Cache domains identified by newly constructed models in the Pfam 27.0 associated UniProt database (June 2012 release). (XLSX)

S3 Data Cache domains identified by newly constructed models in the NCBI non-redundant database (April 2015 release). (XLSX)

S4 Data HMM-HMM comparison of newly identified Cache sequences in NR database against HHpred PDB70 database (September 2015). (XLSX)

S5 Data Overlap of Cache domains identified by new models with other Pfam domains. (XLSX)

S6 Data HMM-HMM results. Spreadsheet 1: HHpred domain prediction for extracellular PAS-like structures. Spreadsheet 2: Results for searches initiated with newly built Cache domain models. Spreadsheet 3: HHsearch all-against-all Cache, PAS, GAF. Spreadsheet 4: HHsearch initiated with new members of the Cache superfamily.
(XLSX)

S7 Data Sequence logos for newly defined Cache superfamily. (PDF)

S8 Data Phyletic distribution of Cache, PAS and GAF domains. (XLSX)
