# Supplementary figures and images for: Cache Domains That are Homologous to, but Different from PAS Domains Comprise the Largest Superfamily of Extracellular Sensors in Prokaryotes
Source: PLoS Comput Biol. 2016 Apr 6;12(4):e1004862. doi: 10.1371/journal.pcbi.1004862 (PMC4822843; doi:10.1371/journal.pcbi.1004862)

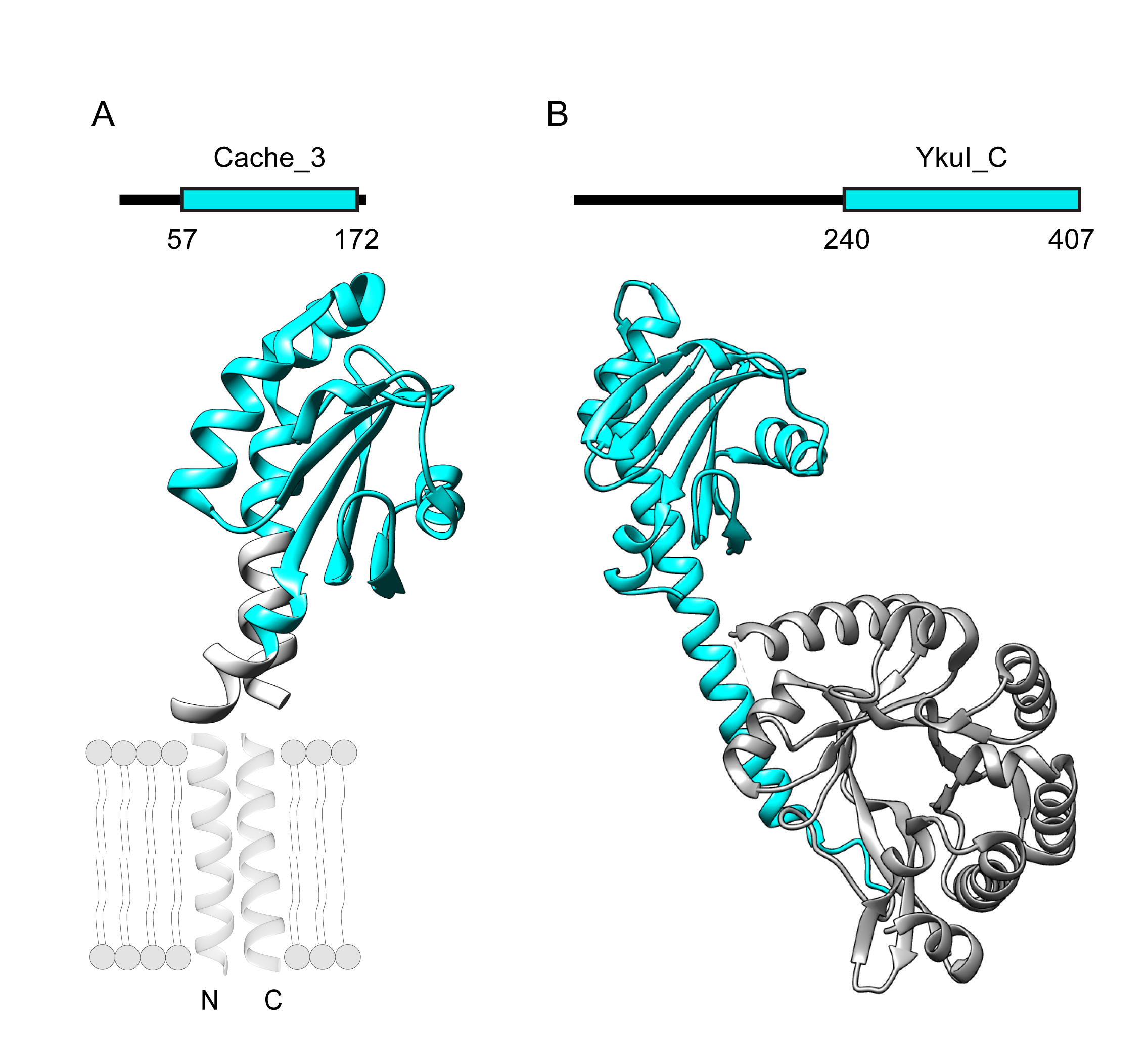

Supplement: S1 Fig — (A) Periplasmic domain of CitA from Klebsiella pneumoniae (PDB-1P0Z). Cache_3 domain is shown in cyan, (B) YkuI comprising of EAL and YkuI_C domains from Bacillus subtilis (PDB-2W27). The EAL domain is shown in gray and YkuI_C domain is shown in cyan. The Pfam based domain predictions for Cache_3 and YkuI_C map to distinct structural domains in contrast to Cache_1 and Cache_2 that map only to parts of structural domains (Fig 2). (TIF) [file pcbi.1004862.s001.tif]

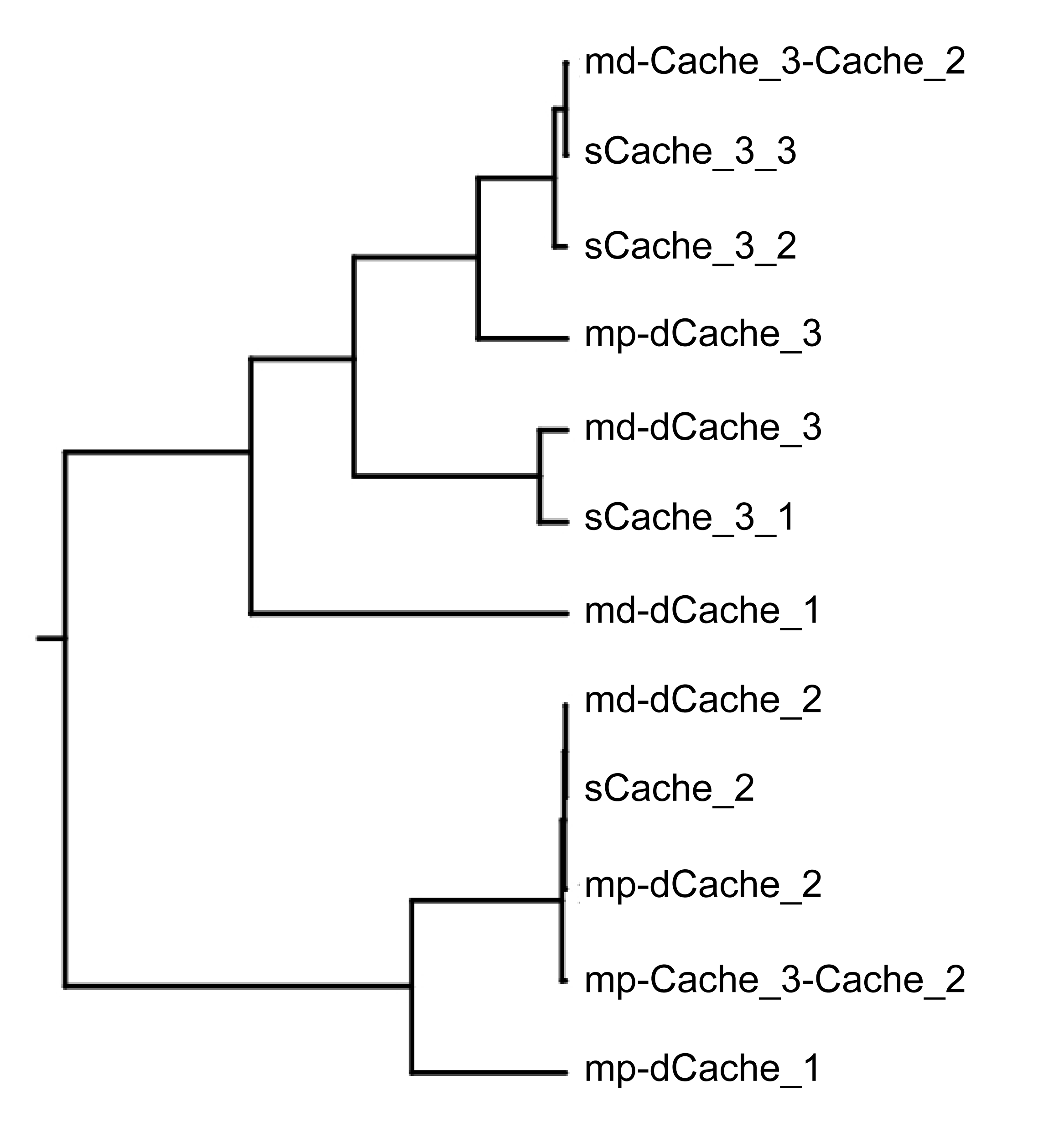

Supplement: S2 Fig — The PAS-like regions were extracted for each model based on secondary structure prediction and all-against-all HHsearch comparison was carried out. The dendrogram was generated by using the probability scores as similarity measure. (TIF) [file pcbi.1004862.s002.tif]

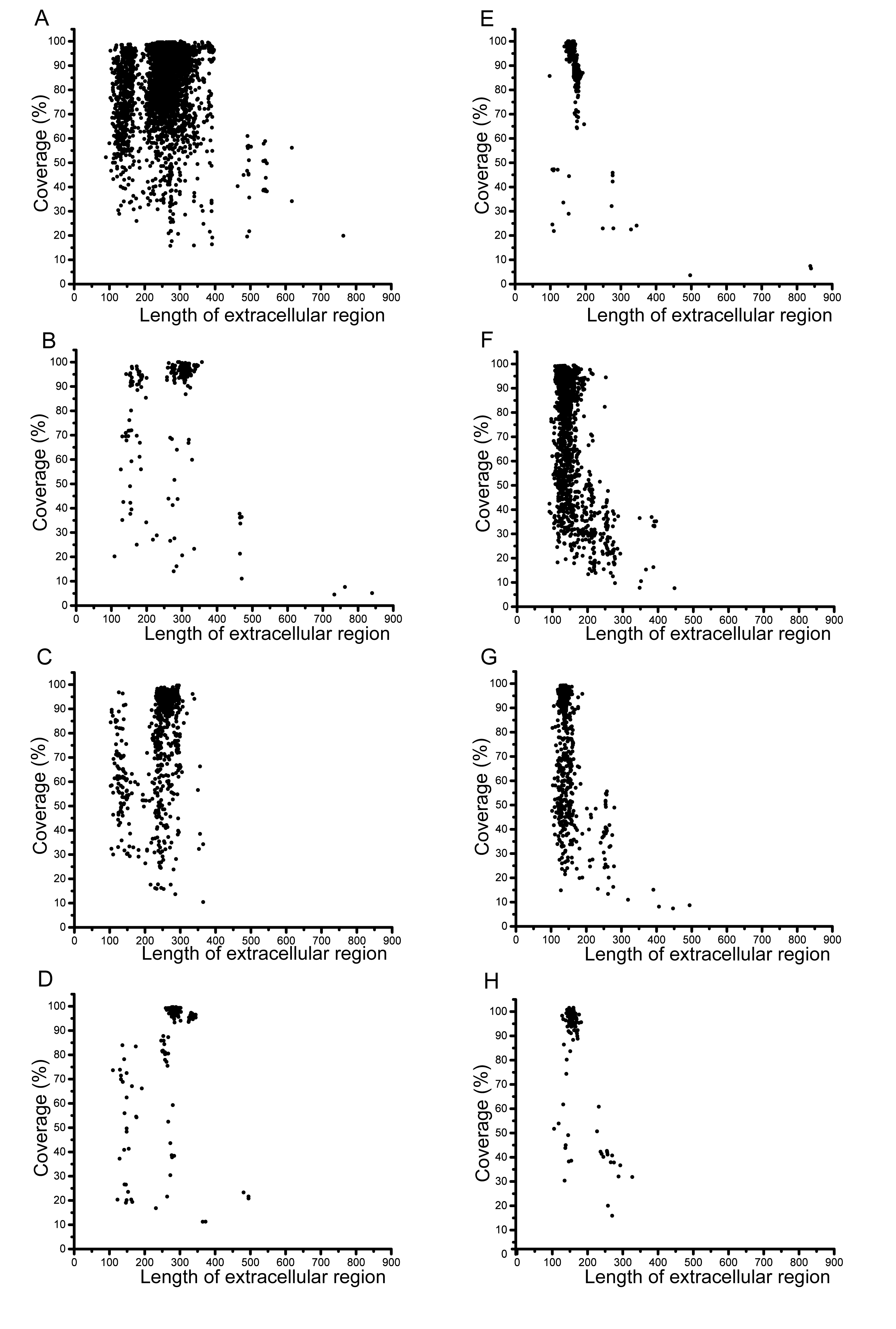

Supplement: S3 Fig — (A) dCache_1, (B) dCacche_2, (C) dCache_3, (D) Cache_3-Cache_2, (E) sCache_2, (F) sCache_3_1, (G) sCache_3_2, (H) sCache_3_3.Scatterplot showing relationship between the length of the extracellular regions and the percent query coverage of the extracellular regions by the new Cache domain models. (TIF) [file pcbi.1004862.s003.tif]

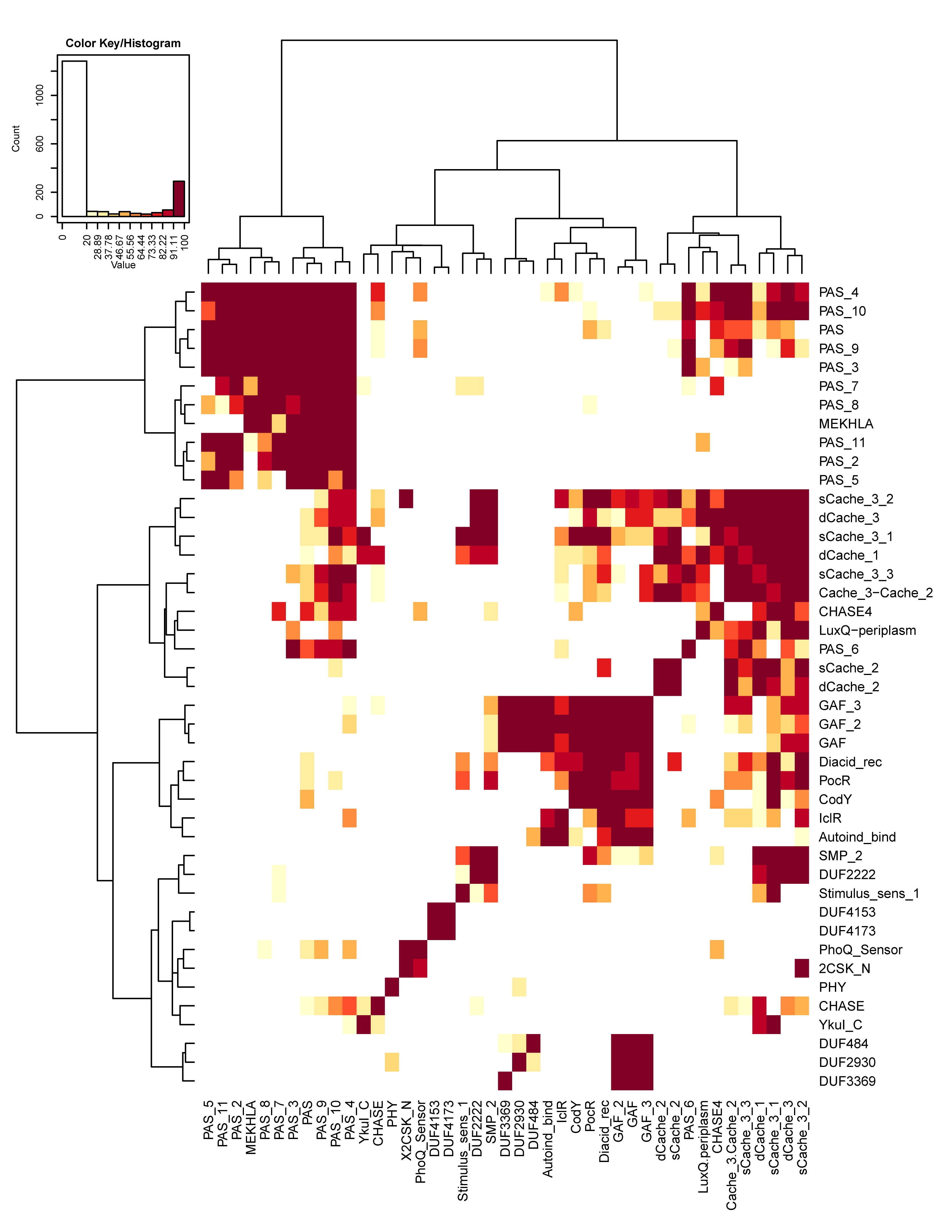

Supplement: S4 Fig — The HHsearch Prob scores were used to generate the heatmap using a threshold Prob score of > = 20, Euclidean distance and Ward clustering using the Heatmap tool http://www.hiv.lanl.gov/content/sequence/HEATMAP/heatmap.html. (TIF) [file pcbi.1004862.s004.tif]

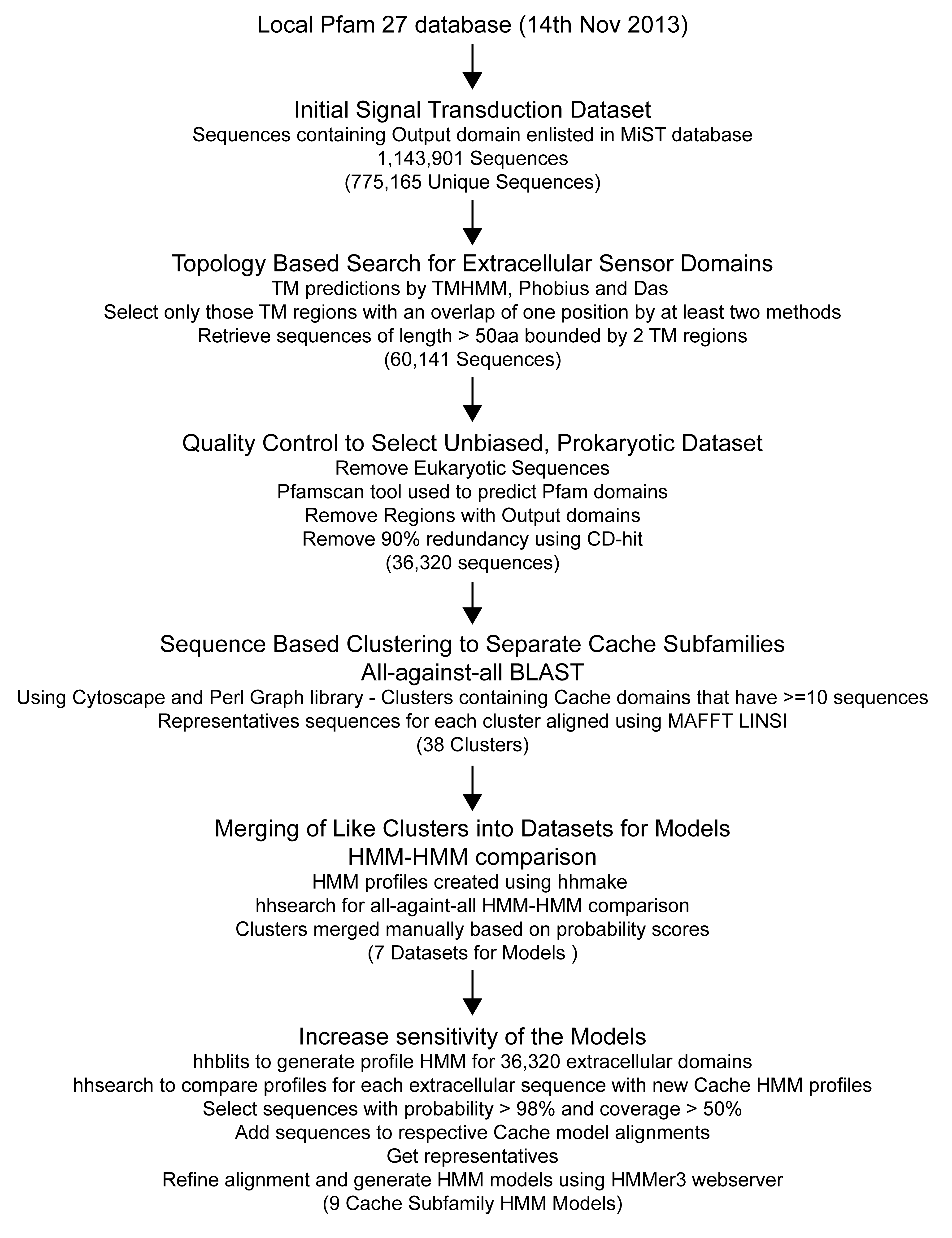

Supplement: S5 Fig — (TIF) [file pcbi.1004862.s005.tif]

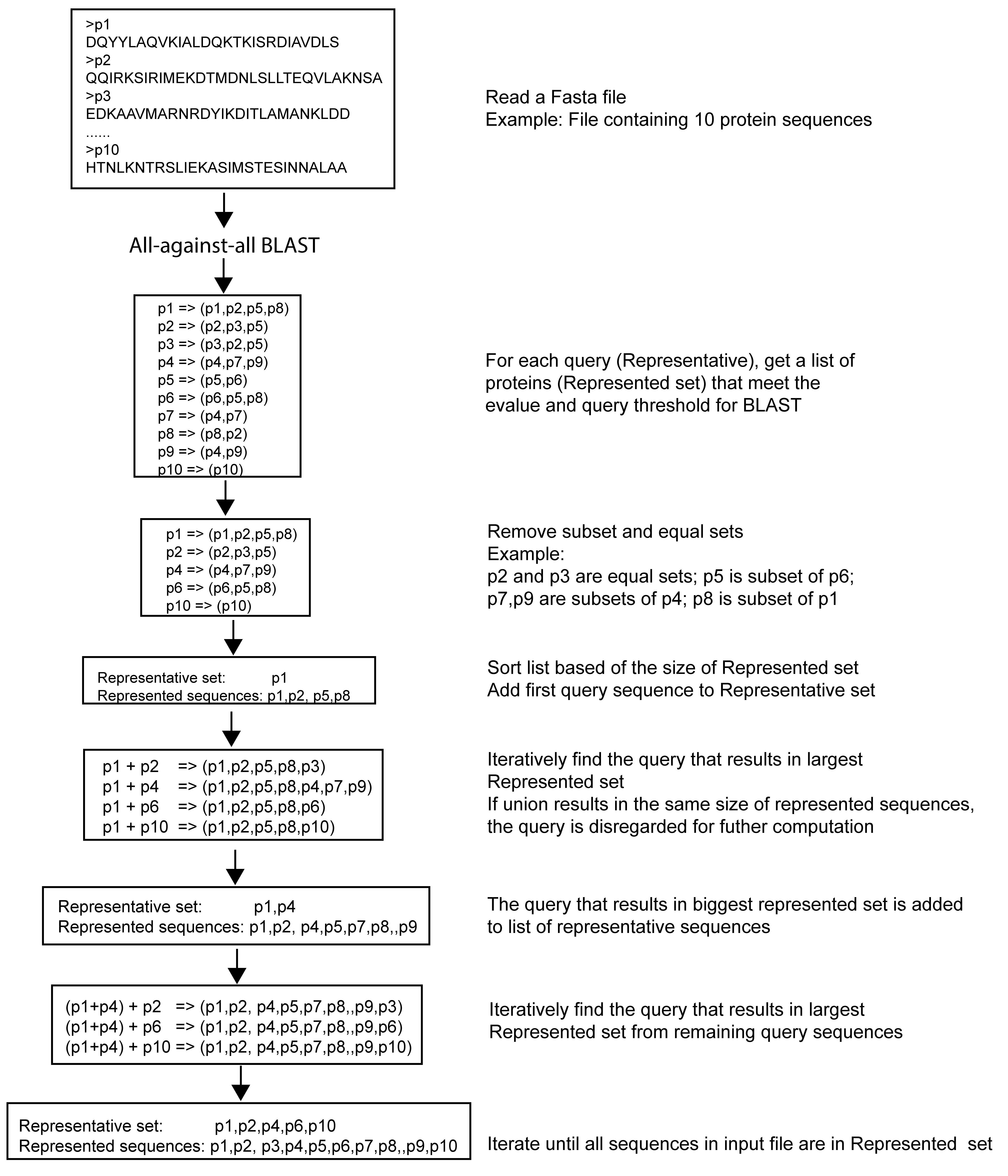

Supplement: S6 Fig — (TIF) [file pcbi.1004862.s006.tif]

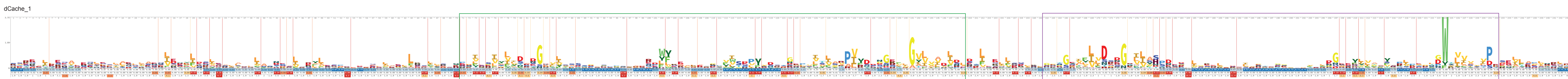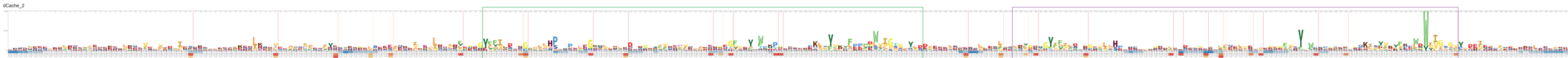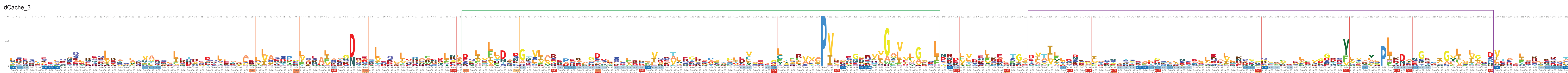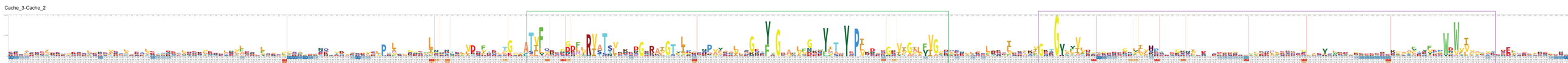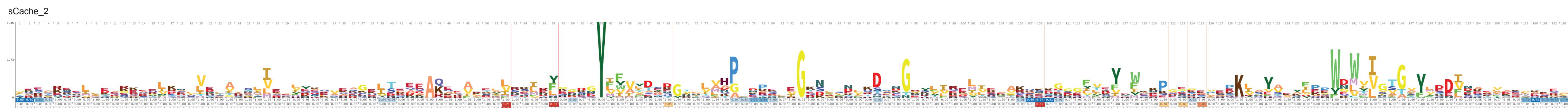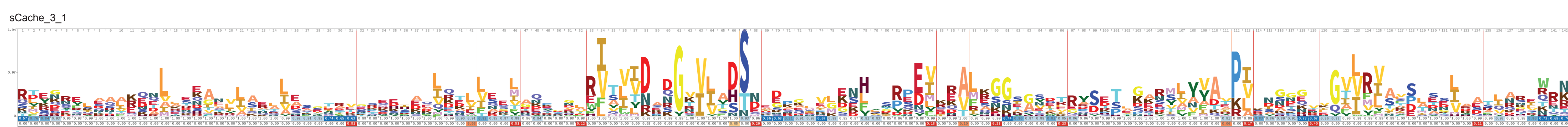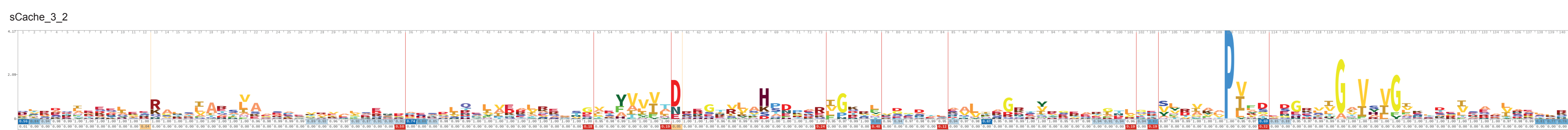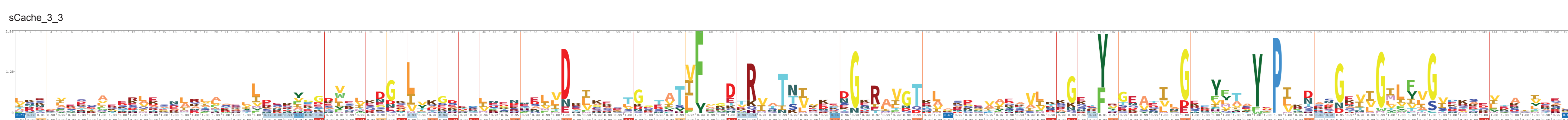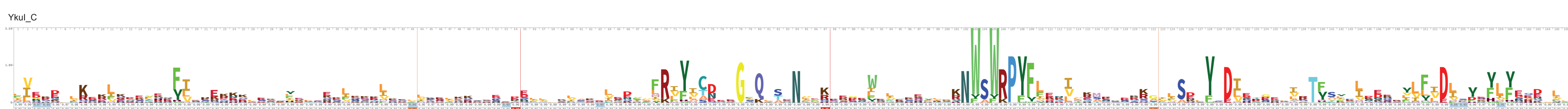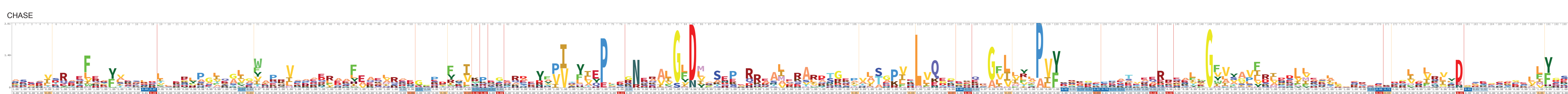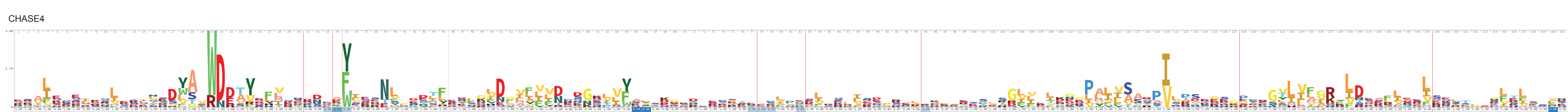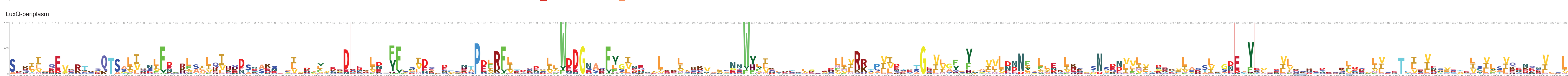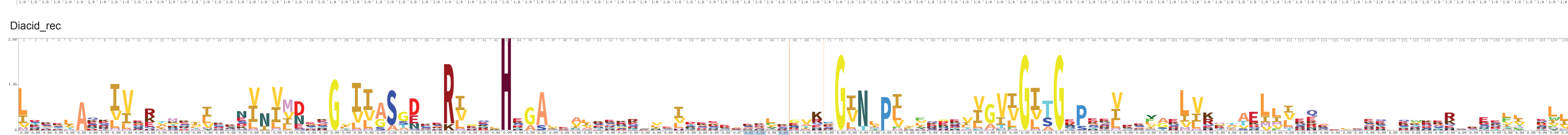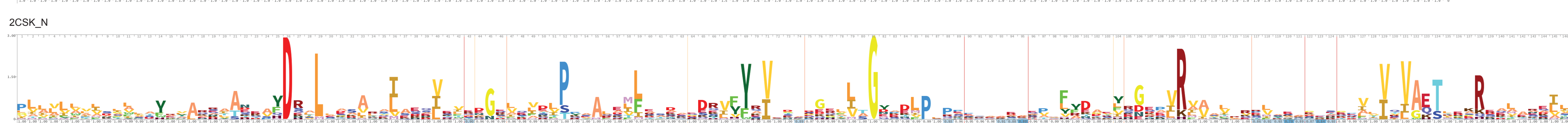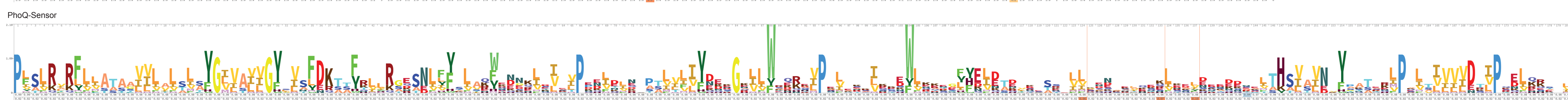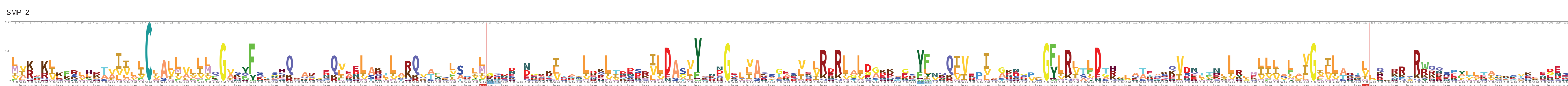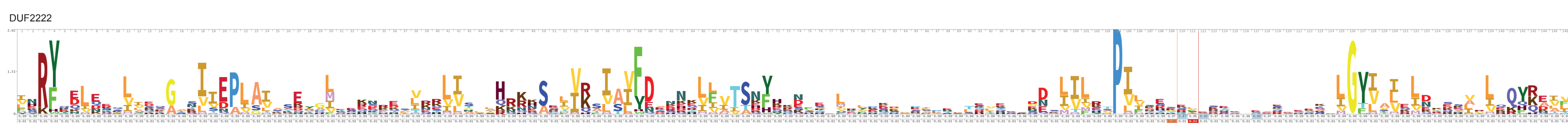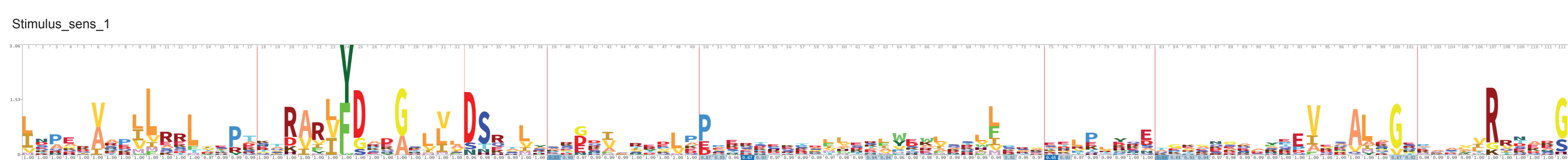

Supplement: S7 Data — (PDF) [file pcbi.1004862.s023.pdf]
