# Supplementary material for: Cache Domains That are Homologous to, but Different from PAS Domains Comprise the Largest Superfamily of Extracellular Sensors in Prokaryotes
Source: PLoS Comput Biol. 2016 Apr 6;12(4):e1004862. doi: 10.1371/journal.pcbi.1004862 (PMC4822843; doi:10.1371/journal.pcbi.1004862)
Supplement: S1 Table — (DOCX) [file pcbi.1004862.s007.docx]

**S1 Table. Timeline of PAS, Cache and PDC domain discoveries.**

| **Year** | **Highlights** | **References** |
| --- | --- | --- |
| 1997 | PAS domain defined | [1, 2] |
| 2000 | Cache domain defined based on sequence similarity  CitA circular permutation of Cache  Cache N-terminal similar to PAS core | [3] |
| 2003 | Structure of CitA solved  Suggested to be PAS based on structural similarity to PYP  No PAS detected by BLAST, 3D-PSSM, LOOPP, manual searching of S1/S2 boxes  First structure for extracytoplasmic PAS | [4] |
| 2003 | Structure of DcuS (belongs to CitA family) solved  Suggested to possess a novel domain | [5] |
| 2008 | Structure of PhoQ solved  New family PDC (PhoQ-DcuS-CitA)  PDC family subset of PAS – difference in N-terminal helix and the region between 2^nd^ and 3^rd^ strands  PDC and PAS belong to separate superfamilies | [6] |
| 2008 | PDC family comprised of single PDC (DcuS) and double PDC (*Vibrio cholerae* DctB) | [7] |
| 2008 | *Sinorhizobium meliloti* DctB comprised of tandem PAS domains and one N-terminal helical region | [8] |
| 2010 | Structures of dPDC proteins solved  PhoQ, CitA, DcuS, AbsF, PhoR – sPDC  LuxQ, DctB, KinD - dPDC | [9] |
| 2013 | CitA and DcuS part of Cache_3, a new family in the Cache clan | [10] |

**References**

1. Ponting CP, Aravind L. PAS: a multifunctional domain family comes to light. Curr Biol. 1997;7(11):R674-7.

2. Zhulin IB, Taylor BL, Dixon R. PAS domain S-boxes in Archaea, Bacteria and sensors for oxygen and redox. Trends Biochem Sci. 1997;22(9):331-3.

3. Anantharaman V, Aravind L. Cache - a signaling domain common to animal Ca(2+)-channel subunits and a class of prokaryotic chemotaxis receptors. Trends Biochem Sci. 2000;25(11):535-7.

4. Reinelt S, Hofmann E, Gerharz T, Bott M, Madden DR. The structure of the periplasmic ligand-binding domain of the sensor kinase CitA reveals the first extracellular PAS domain. J Biol Chem. 2003;278(40):39189-96. doi: 10.1074/jbc.M305864200.

5. Pappalardo L, Janausch IG, Vijayan V, Zientz E, Junker J, Peti W, et al. The NMR structure of the sensory domain of the membranous two-component fumarate sensor (histidine protein kinase) DcuS of Escherichia coli. J Biol Chem. 2003;278(40):39185-8. doi: 10.1074/jbc.C300344200.

6. Cheung J, Bingman CA, Reyngold M, Hendrickson WA, Waldburger CD. Crystal structure of a functional dimer of the PhoQ sensor domain. J Biol Chem. 2008;283(20):13762-70. doi: 10.1074/jbc.M710592200.

7. Cheung J, Hendrickson WA. Crystal structures of C4-dicarboxylate ligand complexes with sensor domains of histidine kinases DcuS and DctB. J Biol Chem. 2008;283(44):30256-65. doi: 10.1074/jbc.M805253200.

8. Zhou YF, Nan B, Nan J, Ma Q, Panjikar S, Liang YH, et al. C4-dicarboxylates sensing mechanism revealed by the crystal structures of DctB sensor domain. J Mol Biol. 2008;383(1):49-61. doi: 10.1016/j.jmb.2008.08.010.

9. Zhang Z, Hendrickson WA. Structural characterization of the predominant family of histidine kinase sensor domains. J Mol Biol. 2010;400(3):335-53. doi: 10.1016/j.jmb.2010.04.049.

10. Finn RD, Bateman A, Clements J, Coggill P, Eberhardt RY, Eddy SR, et al. Pfam: the protein families database. Nucleic Acids Research. 2014;42(D1):D222-D30. doi: 10.1093/nar/gkt1223.
