# Supplementary material for: Cache Domains That are Homologous to, but Different from PAS Domains Comprise the Largest Superfamily of Extracellular Sensors in Prokaryotes
Source: PLoS Comput Biol. 2016 Apr 6;12(4):e1004862. doi: 10.1371/journal.pcbi.1004862 (PMC4822843; doi:10.1371/journal.pcbi.1004862)
Supplement: S2 Table — (DOCX) [file pcbi.1004862.s008.docx]

**S2 Table. Family (domain) and superfamily assignments for extracellular PAS-like domains.**

| **PDB** | **Organism** | **Gene** | **Domain**  **name** | **Refs** | **SCOP superfamily** | **CATH superfamily** | **Pfam family** | **Pfam clan** |
| --- | --- | --- | --- | --- | --- | --- | --- | --- |
| 1P0Z | *Klebsiella pneumoniae* | CitA | PAS  Cache  PDC | [1]  [2]  [3] | Sensory domain-like | 3.30.450.20 | Cache_3 | Cache |
| 3BY8 | *Escherichia coli* | DcuS | PAS-like  Cache  PDC | [4]  [2]  [3] | Sensory domain-like | 3.30.450.20 | Cache_3 | Cache |
| 3C8C | *Vibrio cholerae* | VCA0923 | PAS  PDC | [5]  [6] | N/A | 3.30.450.20 | Cache_1 | Cache |
| 1YAX | *Salmonella enterica* | PhoQ | PAS | [7] | N/A | N/A | PhoQ_Sensor | N/A |
| 3BQ8 | *Escherichia coli* | PhoQ | PDC  PAS | [3]  [5] | N/A | N/A | PhoQ_Sensor | N/A |
| 2HJE | *Vibrio harveyi* | LuxQ | PAS  PDC | [8]  [6] | Sensory domain-like | N/A | LuxQ-periplasm | N/A |
| 3C38 | *Vibrio cholerae* | LuxQ | PAS  PDC | [5]  [6] | Sensory domain-like | N/A | LuxQ-periplasm | N/A |
| 3E4P | *Sinorhizobium meliloti* | DctB | PAS  PDC | [9]  [6] | N/A | N/A | N/A | N/A |
| 3BY9 | *Vibrio cholerae* | DctB | PDC | [10] | N/A | N/A | YkuI_C | Cache |
| 3B42 | *Geobacter sulfurreducens* | GSU0935 | PAS | [11] | N/A | N/A | N/A | N/A |
| 2W27 | *Bacillus subtilis* | YkuI | PAS-like  PAS | [12]  [5] | Sensory domain-like | 3.30.450.20 | YkuI_C | Cache |
| 2VA0 | *Cellvibrio japonicus* | AbsF | PAS  PDC | [13]  [6] | N/A | N/A | N/A | N/A |
| 3LIA | *Methanosarcina mazei* | MM2955 | PDC  PAS | [6]  [5] | N/A | N/A | Cache_1 | Cache |
| 3LIB | *Methanosarcina mazei* | MM2965 | PDC  PAS | [6]  [5] | N/A | N/A | Cache_1 | Cache |
| 3LIC | *Shewanella oneidensis* | SO0859 | PDC  PAS | [6]  [5] | N/A | N/A | N/A | N/A |
| 3LID | *Vibrio parahaemolyticus* | VP0354 | PDC  PAS | [6]  [5] | Sensory domain-like | 3.30.450.20 | N/A | N/A |
| 3LIF | *Rhodopseudomonas palustris* | RPA3616 | PDC  PAS | [6]  [5] | N/A | N/A | N/A | N/A |
| 3CWF | *Bacillus subtilis* | PhoR | PAS-like  PDC  PAS | [14]  [6]  [5] | N/A | 3.30.450.20 | N/A | N/A |
| 3T4J | *Arabidopsis thaliana* | AHK4 | PAS-like  CHASE | [15]  [16] | N/A | N/A | CHASE | N/A |
| 4JGO | *Bacillus subtilis* | KinD | PAS-like  PDC  PAS | [17]  [6]  [5] | N/A | N/A | N/A | N/A |
| 2QHK | *Vibrio parahaemolyticus* | VP0183 | PAS | [5] | N/A | 3.30.450.20 | Cache_2 | Cache |

N/A – not assigned; Pfam clan assignments are based on default E-values, Pfam 27.0 release.

**References**

1. Reinelt S, Hofmann E, Gerharz T, Bott M, Madden DR. The structure of the periplasmic ligand-binding domain of the sensor kinase CitA reveals the first extracellular PAS domain. J Biol Chem. 2003;278(40):39189-96. doi: 10.1074/jbc.M305864200.

2. Anantharaman V, Aravind L. Cache - a signaling domain common to animal Ca(2+)-channel subunits and a class of prokaryotic chemotaxis receptors. Trends Biochem Sci. 2000;25(11):535-7.

3. Cheung J, Bingman CA, Reyngold M, Hendrickson WA, Waldburger CD. Crystal structure of a functional dimer of the PhoQ sensor domain. J Biol Chem. 2008;283(20):13762-70. doi: 10.1074/jbc.M710592200.

4. Pappalardo L, Janausch IG, Vijayan V, Zientz E, Junker J, Peti W, et al. The NMR structure of the sensory domain of the membranous two-component fumarate sensor (histidine protein kinase) DcuS of Escherichia coli. J Biol Chem. 2003;278(40):39185-8. doi: 10.1074/jbc.C300344200.

5. Henry JT, Crosson S. Ligand-binding PAS domains in a genomic, cellular, and structural context. Annu Rev Microbiol. 2011;65:261-86. doi: 10.1146/annurev-micro-121809-151631.

6. Zhang Z, Hendrickson WA. Structural characterization of the predominant family of histidine kinase sensor domains. J Mol Biol. 2010;400(3):335-53. doi: 10.1016/j.jmb.2010.04.049.

7. Cho US, Bader MW, Amaya MF, Daley ME, Klevit RE, Miller SI, et al. Metal bridges between the PhoQ sensor domain and the membrane regulate transmembrane signaling. J Mol Biol. 2006;356(5):1193-206. doi: 10.1016/j.jmb.2005.12.032.

8. Neiditch MB, Federle MJ, Pompeani AJ, Kelly RC, Swem DL, Jeffrey PD, et al. Ligand-induced asymmetry in histidine sensor kinase complex regulates quorum sensing. Cell. 2006;126(6):1095-108. doi: 10.1016/j.cell.2006.07.032.

9. Zhou YF, Nan B, Nan J, Ma Q, Panjikar S, Liang YH, et al. C4-dicarboxylates sensing mechanism revealed by the crystal structures of DctB sensor domain. J Mol Biol. 2008;383(1):49-61. doi: 10.1016/j.jmb.2008.08.010.

10. Cheung J, Hendrickson WA. Crystal structures of C4-dicarboxylate ligand complexes with sensor domains of histidine kinases DcuS and DctB. J Biol Chem. 2008;283(44):30256-65. doi: 10.1074/jbc.M805253200.

11. Pokkuluri PR, Pessanha M, Londer YY, Wood SJ, Duke NE, Wilton R, et al. Structures and solution properties of two novel periplasmic sensor domains with c-type heme from chemotaxis proteins of Geobacter sulfurreducens: implications for signal transduction. J Mol Biol. 2008;377(5):1498-517. doi: 10.1016/j.jmb.2008.01.087.

12. Minasov G, Padavattan S, Shuvalova L, Brunzelle JS, Miller DJ, Basle A, et al. Crystal structures of YkuI and its complex with second messenger cyclic Di-GMP suggest catalytic mechanism of phosphodiester bond cleavage by EAL domains. J Biol Chem. 2009;284(19):13174-84. doi: 10.1074/jbc.M808221200.

13. Emami K, Topakas E, Nagy T, Henshaw J, Jackson KA, Nelson KE, et al. Regulation of the xylan-degrading apparatus of Cellvibrio japonicus by a novel two-component system. J Biol Chem. 2009;284(2):1086-96. doi: 10.1074/jbc.M805100200.

14. Chang C, Tesar C, Gu M, Babnigg G, Joachimiak A, Pokkuluri PR, et al. Extracytoplasmic PAS-like domains are common in signal transduction proteins. J Bacteriol. 2010;192(4):1156-9. doi: 10.1128/JB.01508-09.

15. Hothorn M, Dabi T, Chory J. Structural basis for cytokinin recognition by Arabidopsis thaliana histidine kinase 4. Nat Chem Biol. 2011;7(11):766-8. doi: 10.1038/nchembio.667.

16. Mougel C, Zhulin IB. CHASE: an extracellular sensing domain common to transmembrane receptors from prokaryotes, lower eukaryotes and plants. Trends Biochem Sci. 2001;26(10):582-4.

17. Wu R, Gu M, Wilton R, Babnigg G, Kim Y, Pokkuluri PR, et al. Insight into the sporulation phosphorelay: crystal structure of the sensor domain of Bacillus subtilis histidine kinase, KinD. Protein Sci. 2013;22(5):564-76. doi: 10.1002/pro.2237.
