# Supplementary material for: Cache Domains That are Homologous to, but Different from PAS Domains Comprise the Largest Superfamily of Extracellular Sensors in Prokaryotes
Source: PLoS Comput Biol. 2016 Apr 6;12(4):e1004862. doi: 10.1371/journal.pcbi.1004862 (PMC4822843; doi:10.1371/journal.pcbi.1004862)
Supplement: S3 Table — (DOCX) [file pcbi.1004862.s009.docx]

**S3 Table. Family (domain) and superfamily assignments for intracellular PAS domains.**

| **PDB** | **Organism** | **Gene** | **Domain**  **name** | **Refs** | **SCOP superfamily** | **CATH superfamily** | **Pfam clan** |
| --- | --- | --- | --- | --- | --- | --- | --- |
| 1NWZ | *Ectothiorhodospira halophila* | PYP | PAS | [1] | PYP-like sensor (PAS domain) | 3.30.450.20 | PAS |
| 3F1P | *Homo sapiens* | Hif2a | PAS | [2] | PYP-like sensor (PAS domain) | 3.30.450.20 | PAS |
| 1S67 | *Escherichia coli* | DosP | PAS | [3] | PYP-like sensor (PAS domain) | 3.30.450.20 | PAS |
| 1D06 | *Rhizobium meliloti* | FixL | PAS | [4] | PYP-like sensor (PAS domain) | 3.30.450.20 | PAS |
| 1MZU | *Rhodospirillum centenum* | Pph | PAS | [5] | PYP-like sensor (PAS domain) | 3.30.450.20 | PAS |
| 1N9L | *Chlamydomonas reinhardtii* | Phot | LOV | [6] | PYP-like sensor (PAS domain) | 3.30.450.20 | PAS |
| 1OJ5 | *Mus musculus* | NCOA1 | PAS | [7] | PYP-like sensor (PAS domain) | 3.30.450.20 | PAS |
| 3KX0 | *Mycobacterium tuberculosis* | Rv1364c | PAS | [8] | N/A | 3.30.450.20 | PAS |
| 1LL8 | *Homo sapiens* | KIAA0135 | PAS | [9] | PYP-like sensor (PAS domain) | 3.30.450.20 | PAS |
| 3GDI | *Mus musculus* | Per2 | PAS | [10] | N/A | 3.30.450.20 | PAS |
| 1BYW | *Homo sapiens* | HERG | PAS | [11] | PYP-like sensor (PAS domain) | 3.30.450.20 | PAS |
| 2VEA | *Synechocystis sp. PCC6803* | Cph1 | PAS | [12] | PYP-like sensor (PAS domain) | N/A | PAS |
| 1JNU | *Adiantum capilus-veneris* | PHY3 | LOV | [13] | PYP-like sensor (PAS domain) | 3.30.450.20 | PAS |
| 3C2W | *Pseudomonas aeruginosa* | BhpP | PAS | [14] | PYP-like sensor (PAS domain) | N/A | PAS |
| 3RH8 | *Neurospora crassa* | vvd | LOV | [15] | N/A | N/A | PAS |
| 4OUR | *Arabidopsis thaliana* | PHYB | PAS | [16] | N/A | N/A | PAS |
| 3A0S | *Thermotoga maritima* | TM_1359 | PAS | [17] | N/A | 3.30.450.20 | PAS |
| 3BWL | *Haloarcula marismortui* | HtlD | N/A |  | N/A | 3.30.450.20 | PAS |
| 1X0O | *Homo sapiens* | BHLHE2 | PAS | [18] | N/A | 3.30.450.20 | PAS |
| 2YKH | *Mycobacterium tuberculosis* | Rv3220c | PAS | [19] | N/A | N/A | N/A |
| 4HIA | *Rhodobacter sphaeroides* |  | LOV | [20] | N/A | N/A | PAS |
| 3EWK | *Methylococcus capsulatus* | MmoS | PAS | [21] | PYP-like sensor (PAS domain) | 3.30.450.20 | PAS |

N/A – not assigned; LOV, a subfamily of the PAS domain

**References**

1. Getzoff ED, Gutwin KN, Genick UK. Anticipatory active-site motions and chromophore distortion prime photoreceptor PYP for light activation. Nat Struct Biol. 2003;10(8):663-8. doi: 10.1038/nsb958.

2. Scheuermann TH, Tomchick DR, Machius M, Guo Y, Bruick RK, Gardner KH. Artificial ligand binding within the HIF2alpha PAS-B domain of the HIF2 transcription factor. Proc Natl Acad Sci U S A. 2009;106(2):450-5. doi: 10.1073/pnas.0808092106.

3. Park H, Suquet C, Satterlee JD, Kang C. Insights into signal transduction involving PAS domain oxygen-sensing heme proteins from the X-ray crystal structure of Escherichia coli Dos heme domain (Ec DosH). Biochemistry. 2004;43(10):2738-46. doi: 10.1021/bi035980p.

4. Miyatake H, Mukai M, Park SY, Adachi S, Tamura K, Nakamura H, et al. Sensory mechanism of oxygen sensor FixL from Rhizobium meliloti: crystallographic, mutagenesis and resonance Raman spectroscopic studies. J Mol Biol. 2000;301(2):415-31. doi: 10.1006/jmbi.2000.3954.

5. Rajagopal S, Moffat K. Crystal structure of a photoactive yellow protein from a sensor histidine kinase: conformational variability and signal transduction. Proc Natl Acad Sci U S A. 2003;100(4):1649-54. doi: 10.1073/pnas.0336353100.

6. Fedorov R, Schlichting I, Hartmann E, Domratcheva T, Fuhrmann M, Hegemann P. Crystal structures and molecular mechanism of a light-induced signaling switch: The Phot-LOV1 domain from Chlamydomonas reinhardtii. Biophys J. 2003;84(4):2474-82. doi: 10.1016/S0006-3495(03)75052-8.

7. Razeto A, Ramakrishnan V, Litterst CM, Giller K, Griesinger C, Carlomagno T, et al. Structure of the NCoA-1/SRC-1 PAS-B domain bound to the LXXLL motif of the STAT6 transactivation domain. J Mol Biol. 2004;336(2):319-29.

8. Jaiswal RK, Manjeera G, Gopal B. Role of a PAS sensor domain in the Mycobacterium tuberculosis transcription regulator Rv1364c. Biochem Biophys Res Commun. 2010;398(3):342-9. doi: 10.1016/j.bbrc.2010.06.027.

9. Amezcua CA, Harper SM, Rutter J, Gardner KH. Structure and interactions of PAS kinase N-terminal PAS domain: model for intramolecular kinase regulation. Structure. 2002;10(10):1349-61.

10. Hennig S, Strauss HM, Vanselow K, Yildiz O, Schulze S, Arens J, et al. Structural and functional analyses of PAS domain interactions of the clock proteins Drosophila PERIOD and mouse PERIOD2. PLoS Biol. 2009;7(4):e94. doi: 10.1371/journal.pbio.1000094.

11. Morais Cabral JH, Lee A, Cohen SL, Chait BT, Li M, Mackinnon R. Crystal structure and functional analysis of the HERG potassium channel N terminus: a eukaryotic PAS domain. Cell. 1998;95(5):649-55.

12. Essen LO, Mailliet J, Hughes J. The structure of a complete phytochrome sensory module in the Pr ground state. Proc Natl Acad Sci U S A. 2008;105(38):14709-14. doi: 10.1073/pnas.0806477105.

13. Crosson S, Moffat K. Photoexcited structure of a plant photoreceptor domain reveals a light-driven molecular switch. Plant Cell. 2002;14(5):1067-75.

14. Yang X, Kuk J, Moffat K. Crystal structure of Pseudomonas aeruginosa bacteriophytochrome: photoconversion and signal transduction. Proc Natl Acad Sci U S A. 2008;105(38):14715-20. doi: 10.1073/pnas.0806718105.

15. Vaidya AT, Chen CH, Dunlap JC, Loros JJ, Crane BR. Structure of a light-activated LOV protein dimer that regulates transcription. Sci Signal. 2011;4(184):ra50. doi: 10.1126/scisignal.2001945.

16. Burgie ES, Bussell AN, Walker JM, Dubiel K, Vierstra RD. Crystal structure of the photosensing module from a red/far-red light-absorbing plant phytochrome. Proc Natl Acad Sci U S A. 2014;111(28):10179-84. doi: 10.1073/pnas.1403096111.

17. Yamada S, Sugimoto H, Kobayashi M, Ohno A, Nakamura H, Shiro Y. Structure of PAS-linked histidine kinase and the response regulator complex. Structure. 2009;17(10):1333-44. doi: 10.1016/j.str.2009.07.016.

18. Card PB, Erbel PJ, Gardner KH. Structural basis of ARNT PAS-B dimerization: use of a common beta-sheet interface for hetero- and homodimerization. J Mol Biol. 2005;353(3):664-77. doi: 10.1016/j.jmb.2005.08.043.

19. Preu J, Panjikar S, Morth P, Jaiswal R, Karunakar P, Tucker PA. The sensor region of the ubiquitous cytosolic sensor kinase, PdtaS, contains PAS and GAF domain sensing modules. J Struct Biol. 2012;177(2):498-505. doi: 10.1016/j.jsb.2011.11.012.

20. Conrad KS, Bilwes AM, Crane BR. Light-induced subunit dissociation by a light-oxygen-voltage domain photoreceptor from Rhodobacter sphaeroides. Biochemistry. 2013;52(2):378-91. doi: 10.1021/bi3015373.

21. Ukaegbu UE, Rosenzweig AC. Structure of the redox sensor domain of Methylococcus capsulatus (Bath) MmoS. Biochemistry. 2009;48(10):2207-15. doi: 10.1021/bi8019614.
