# Supplementary material for: Cache Domains That are Homologous to, but Different from PAS Domains Comprise the Largest Superfamily of Extracellular Sensors in Prokaryotes
Source: PLoS Comput Biol. 2016 Apr 6;12(4):e1004862. doi: 10.1371/journal.pcbi.1004862 (PMC4822843; doi:10.1371/journal.pcbi.1004862)
Supplement: S4 Table — (DOCX) [file pcbi.1004862.s010.docx]

**S4 Table. Best Pfam database matches for extracellular PAS-like domains in sequence-profile and profile-profile searches.**

| **PDB** | **Organism** | **HMMer (hmmscan)** | | | **HHpred** | | |
| --- | --- | --- | --- | --- | --- | --- | --- |
|  |  | **Best hit** | **E-value** | **Clan** | **Best hit** | **Probability%** | **Clan** |
| 1P0Z | *Klebsiella pneumoniae* | Cache_3 | 4.9e-34 | Cache | Cache_3 | 99.7 | Cache |
| 3BY8 | *Escherichia coli* | Cache_3 | 6.2e-38 | Cache | Cache_3 | 99.9 | Cache |
| 3C8C | *Vibrio cholerae* | MCP_N  Cache_1 | 3.9e-32  3.6e-21 | Cache | MCP_N  Cache_1 | 98  99.8 | Cache |
| 1YAX | *Salmonella enterica* | PhoQ_  sensor | 1.6e-69 | N/A | PhoQ_  sensor | 100 | N/A |
| 3BQ8 | *Escherichia coli* | PhoQ_  sensor | 2.9e-69 | N/A | PhoQ_  sensor | 100 | N/A |
| 2HJE | *Vibrio harveyi* | LuxQ-periplasm | 6.5e-109 | N/A | LuxQ-periplasm | 100 | N/A |
| 3C38 | *Vibrio cholerae* | LuxQ-periplasm | 6.6e-106 | N/A | LuxQ-periplasm | 100 | N/A |
| 3E4P | *Sinorhizobium meliloti* | Cache_3 | 0.83 | Cache | Cache_1 | 99.4 | Cache |
| 3BY9 | *Vibrio cholerae* | YkuI_C | 4.1e-5 | Cache | Cache_1 | 99.5 | Cache |
| 3B42 | *Geobacter sulfurreducens* | DUF3365 | 0.984 | N/A | DUF3365 | 99.3 | N/A |
| 2W27 | *Bacillus subtilis* | YkuI_C | 2.2e-73 | Cache | YkuI_C | 100 | Cache |
| 2VA0 | *Cellvibrio japonicus* | no hit |  | N/A | DUF2286 | 74.2 | N/A |
| 3LIA | *Methanosarcina mazei* | Cache_1 | 3.6e-13 | Cache | Cache_1 | 99.7 | Cache |
| 3LIB | *Methanosarcina mazei* | Cache_1 | 2.5e-13 | Cache | Cache_1 | 99.7 | Cache |
| 3LIC | *Shewanella oneidensis* | Cache_1 | 1.3e-3 | Cache | Cache_1 | 99.7 | Cache |
| 3LID | *Vibrio parahaemolyticus* | no hit |  | N/A | Cache_1 | 99.6 | Cache |
| 3LIF | *Rhodopseudomonas palustris* | Cache_1 | 3.1e-3 | Cache | Cache_1 | 99.7 | Cache |
| 3CWF | *Bacillus subtilis* | no hit |  | N/A | Cache_3 | 98.6 | Cache |
| 3T4J | *Arabidopsis thaliana* | CHASE | 1.4e-21 | N/A | CHASE | 100 | N/A |
| 4JGO | *Bacillus subtilis* | Cache_1 | 5.4e-3 | Cache | Cache_1 | 99.6 | Cache |
| 2QHK | *Vibrio parahaemolyticus* | Cache_2 | 5.6e-27 | Cache | Cache_2 | 99.5 | Cache |

N/A – not assigned
