# Supplementary material for: Cache Domains That are Homologous to, but Different from PAS Domains Comprise the Largest Superfamily of Extracellular Sensors in Prokaryotes
Source: PLoS Comput Biol. 2016 Apr 6;12(4):e1004862. doi: 10.1371/journal.pcbi.1004862 (PMC4822843; doi:10.1371/journal.pcbi.1004862)
Supplement: S5 Table — (DOCX) [file pcbi.1004862.s011.docx]

**S5 Table. Number of Cache domains predicted by Pfam 27 Cache models and new models against Pfam 27 associated UniProt database (June 2012 release) and NCBI non-redundant (NR) database (April 2015 release)**

| **Models** | **Family** | **Pfam 27** | **NR** |
| --- | --- | --- | --- |
| New models | dCache_1 | 15569 | 60390 |
|  | dCache_2 | 299 | 995 |
|  | dCache_3 | 883 | 2706 |
|  | Cache_3-Cache_2 | 407 | 1733 |
|  | sCache_2 | 2243 | 8043 |
|  | sCache_3_1 | 2854 | 6493 |
|  | sCache_3_2 | 2499 | 7979 |
|  | sCache_3_3 | 276 | 1038 |
|  | **Total** | **25030** | **89377** |
| Pfam 27 models | Cache_1 | 5381 | 18940 |
|  | Cache_2 | 2250 | 7705 |
|  | Cache_3 | 2608 | 8265 |
|  | **Total** | **10239** | **34910** |
