# Supplementary material for: Cache Domains That are Homologous to, but Different from PAS Domains Comprise the Largest Superfamily of Extracellular Sensors in Prokaryotes
Source: PLoS Comput Biol. 2016 Apr 6;12(4):e1004862. doi: 10.1371/journal.pcbi.1004862 (PMC4822843; doi:10.1371/journal.pcbi.1004862)
Supplement: S6 Table — The query coverage was determined by dividing the length of predicted Cache domain over the length of the extracellular region. The frequency distribution table shows the percentage of Cache domains for different query coverage intervals (bin = 10). (DOCX) [file pcbi.1004862.s012.docx]

**S6 Table. Query coverage of extracellular regions by new Cache domain models.** The query coverage was determined by dividing the length of predicted Cache domain over the length of the extracellular region. The frequency distribution table shows the percentage of Cache domains for different query coverage intervals (bin=10).

| **Query Coverage of Extracellular region (%)** | **Percent of Cache domains** |
| --- | --- |
| 100 | 65.27 |
| 90 | 13.8 |
| 80 | 7.02 |
| 70 | 4.86 |
| 60 | 3.06 |
| 50 | 2.71 |
| 40 | 1.81 |
| 30 | 1.04 |
| 20 | 0.38 |
| 10 | 0.06 |
