# Supplementary material for: Cache Domains That are Homologous to, but Different from PAS Domains Comprise the Largest Superfamily of Extracellular Sensors in Prokaryotes
Source: PLoS Comput Biol. 2016 Apr 6;12(4):e1004862. doi: 10.1371/journal.pcbi.1004862 (PMC4822843; doi:10.1371/journal.pcbi.1004862)
Supplement: S7 Table — The query coverage was determined by dividing the length of predicted Cache domain over the length of the extracellular region for the PDB sequence. (DOCX) [file pcbi.1004862.s013.docx]

**S7 Table. Computational coverage of Cache domains in proteins with known 3D structure.** The query coverage was determined by dividing the length of predicted Cache domain over the length of the extracellular region for the PDB sequence.

| **PDB** | **Length of Extracellular region** | **Length of PDB chain** | **Pfam models** | | | | **New models** | | | |
| --- | --- | --- | --- | --- | --- | --- | --- | --- | --- | --- |
|  |  |  | **Domain** | **Start** | **End** | **Coverage** | **Domain** | **Start** | **End** | **Coverage** |
| 2QHK | 173 | 174 | Cache_2 | 14 | 102 | 51.45 | sCache_2 | 14 | 160 | 84.97 |
| 1P0Z | 136 | 131 | Cache_3 | 15 | 127 | 83.09 | sCache_3_2 | 3 | 128 | 92.65 |
| 3BY8 | 142 | 142 | Cache_3 | 18 | 133 | 81.69 | sCache_3_2 | 5 | 142 | 97.18 |
| 3CWF | 121 | 122 | - | - | - | 0 | sCache_3_1 | 8 | 113 | 87.60 |
| 3BY9 | 259 | 260 | YkuI_C | 71 | 114 | 16.99 | dCache_1 | 5 | 215 | 81.47 |
| 3C8C | 274 | 240 | MCP_N | 2 | 70 | 25.18 | dCache_1 | 5 | 237 | 85.04 |
|  |  |  | Cache_1 | 106 | 182 | 32.08 |  |  |  |  |
| 3LI8 | 277 | 291 | Cache_1 | 140 | 207 | 24.55 | dCache_1 | 9 | 279 | 97.83 |
| 3LIB | 278 | 290 | Cache_1 | 138 | 207 | 24.14 | dCache_1 | 9 | 278 | 97.12 |
| 3LIC | 278 | 274 | - | - | - | 0 | dCache_1 | 10 | 269 | 93.53 |
| 3LID | 293 | 295 | - | - | - | 0 | dCache_1 | 12 | 272 | 89.08 |
| 3LIF | 256 | 254 | - | - | - | 0 | dCache_1 | 9 | 249 | 94.14 |
| 4JGO | 215 | 217 | - | - | - | 0 | dCache_1 | 14 | 182 | 78.60 |
| 3E4P | 275 | 305 | - | - | - | 0 | dCache_1 | 53 | 240 | 68.36 |
