# Supplementary material for: Cache Domains That are Homologous to, but Different from PAS Domains Comprise the Largest Superfamily of Extracellular Sensors in Prokaryotes
Source: PLoS Comput Biol. 2016 Apr 6;12(4):e1004862. doi: 10.1371/journal.pcbi.1004862 (PMC4822843; doi:10.1371/journal.pcbi.1004862)
Supplement: S8 Table — (DOCX) [file pcbi.1004862.s014.docx]

**S8 Table. Known ligands for members of the Cache superfamily**

| **Cache Domain** | **Protein** | **Organism** | **Uniprot** | **Ligands** | **Reference(s)** |
| --- | --- | --- | --- | --- | --- |
| dCache_1 | McpU | *Sinorhizobium meliloti* | F7XA01 | Proline, Histidine, Lysine | [[1](#_ENREF_1), [2](#_ENREF_2)] |
|  | CcaA (Tlp1/ Cj1506c) | *Campylobacter jejuni* | Q0P8B2 | Aspartate | [3] |
|  | CcmL  (Tlp3/Cj1564) | *Campylobacter jejuni* | Q0P864 | Attractants - Isoleucine, Purine, Malic acid, Fumaric acid; Repellants – Arginine, Lysine, Glucosamine, Succinic Acid, Thiamine | [[3](#_ENREF_3)] |
|  | McpB | *Bacillus subtilis* | P39215 | Asparagine; supports Aspartate, Glutamine, Histidine | [5] |
|  | McpC | *Bacillus subtilis* | P54576 | Direct: Cysteine, Proline, Threonine, Glycine, Serine, Valine, Alanine, Tyrosine, Phenylalanine, Leucine, Tryptophan, Isoleucine (weak); Indirect via Transporters: ArtP (Arginine, Lysine), GlnH (Glutamine), MetQ (Methionine), YckB (Tryptophan) | [6] |
|  | KinD | *Bacillus subtilis* | O31671 | Pyruvate, Propionate, Butyrate; Glycerol & Manganese | [7,8] |
|  | PctA | *Pseudomonas aeruginosa* | G3XD24 | All 20 amino acids except – Aspartate, Glutamine | [9] |
|  | PctB | *Pseudomonas aeruginosa* | Q9HW91 | Alanine, Arginine, Glutamate, Lysine, Methionine, Tyrosine, Glutamine | [9] |
|  | PctC | *Pseudomonas aeruginosa* | Q9HW93 | Histidine, Proline, GABA | [10] |
|  | PscA (Psa_14525) | *Pseudomonas syringae pv. actinidiae* | - | D-Aspartate, L-Aspartate, Glutamate | [11] |
|  | PscB (Psa_08785) | *Pseudomonas syringae pv. actinidiae* | - | Alanine, Asparagine, Glutamine, Methionine, Isoleucine, Leucine, Phenylalanine, Serine, Tryptophan, L-Homoserine | [11] |
|  | PscC (Psa_18055) | *Pseudomonas syringae pv. actinidiae* | - | Isoleucine, Proline, GABA | [11] |
|  | VfcA  (VF_0777) | *Vibrio fischeri* | Q5E6S4 | Serine, Alanine, Cysteine, Threonine | [12] |
|  | Mlp24 (Vc2161) | *Vibrio cholerae* | Q9KQ43 | Serine, Glycine, Alanine, Cysteine, Arginine, Asparagine, Histidine, Threonine, Lysine, Glutamine, Proline | [13] |
|  | Mlp37(VCA0923) | *Vibrio cholerae* | Q9KL26 | Serine | [13] |
|  | DctB | *Vibrio cholerae* | Q9KQS3 | Succinate | [22,23] |
| dCache_2 | No information available. | | | | |
| dCache_3 | No information available. | | | | |
| Cache_3-Cache_2 | No information available. | | | | |
| sCache_2 | Adeh_3718 | *Anaeromyxobacter dehalogenans 2CP-C* | Q2IFX2 | Acetate | [14] |
|  | TlpB | *Helicobacter pylori* | B6JPK4 | Urea; pH-Sensing | [15] |
|  | PA2652 | *Pseudomonas aeruginosa* | Q9I0I6 | Malate | [16] |
|  | VP0183 | *Vibrio parahaemolyticus* | Q87T87 | Pyruvate | PDB: 4EXO (2QHK) |
| sCache_3_1 | PhoR | *Escherichia coli* | P08400 | Unknown | [17] |
| sCache_3_2 | CitA | *Klebsiella pneumoniae* | P52687 | Citrate | [18] |
|  | DcuS | *Escherichia coli* | P0AEC8 | Fumarate, Malate, Succinate, Tartarate | [19] |
|  | MalK (YufL) | *Bacillus subtilis* | O05250 | Malate | [20] |
|  | CitS | *Bacillus subtilis* | O34427 | Citrate | [21] |
| sCache_3_3 | No information available. | | | | |
| CHASE | AHK4 | *Arabidopsis thaliana* | Q9C5U0 | Cytokinin Hormones | [24] |
| CHASE4 | No information available. | | | | |
| 2CSK_N | QseC | *Escherichia coli (Enterohemorrhagic O157:H7)* | Q8X524 | Autoinducer 3, Adrenergic Hormones (Epinephrine, Norepinephrine) | [25] |
| PhoQ_sensor | PhoQ | *Escherichia coli* | P23837 | Mg^2+^, Ca^2+^ | [26] |
| Stimulus_sens_1 | BvrS (ChvG/ExoS) | *Brucella abortus* | Q57AG8 | Unknown. Shown to interact with ExoR (Sel1 repeats) | [27] |
| LuxQ-periplasm | LuxQ | *Vibrio harveyi* | P54302 | Unknown. Interacts with LuxP and Autoinducer-2. | [28] |
| DUF2222 | BarA | *Escherichia coli* | P0AEC5 | Unknown | [29,30] |
| SMP_2 | AhpA | *Pasteurella multocida* | Q9L8J3 | Unknown | [31] |
| Diacid_rec | CdaR (SdaR/YaeG) | *Escherichia coli* | P37047 | Unknown | [32] |
| YkuI_C | YkuI | *Bacillus subtilis* | O35014 | Unknown | [33] |

**References**

1. Meier VM, Muschler P, Scharf BE. Functional analysis of nine putative chemoreceptor proteins in Sinorhizobium meliloti. J Bacteriol. 2007;189(5): 1816-1826.
2. Webb BA, Hildreth S, Helm RF, Scharf BE. Sinorhizobium meliloti chemoreceptor McpU mediates chemotaxis towards host plant exudates through direct proline sensing. Appl Environ Microbiol. 2014;80(11): 3404-3415.
3. Hartley-Tassell LE, Shewell LK, Day CJ, Wilson JC, Sandhu R, Ketley JM, et al. Identification and characterization of the aspartate chemosensory receptor of Campylobacter jejuni. Mol Microbiol. 2010;75(3): 710-730.
4. Rahman H, King RM, Shewell LK, Semchenko EA, Hartley-Tassell LE, Wilson JC, et al. Characterisation of a multi-ligand binding chemoreceptor CcmL (Tlp3) of Campylobacter jejuni. PLoS Pathog. 2014;10(1): e1003822.
5. Glekas GD, Foster RM, Cates JR, Estrella JA, Wawrzyniak MJ, Rao CV, et al. A PAS domain binds asparagine in the chemotaxis receptor McpB in Bacillus subtilis. J Biol Chem. 2010;285(3): 1870-1878.
6. Glekas GD, Mulhern BJ, Kroc A, Duelfer KA, Lei V, Rao CV, et al. The Bacillus subtilis Chemoreceptor McpC Senses Multiple Ligands Using Two Discrete Mechanisms. J Biol Chem. 2012;287(47): 39412-39418.
7. Shemesh M, Chai Y. A combination of glycerol and manganese promotes biofilm formation in Bacillus subtilis via the histidine kinase KinD signaling. J Bacteriol. 2013;195(12): 2747-2754.
8. Wu R, Gu M, Wilton R, Babnigg G, Kim Y, Pokkuluri P, et al. Insight into the sporulation phosphorelay: Crystal structure of the sensor domain of Bacillus subtilis histidine kinase, KinD. Protein Sci. 2013;22(5): 564-576.
9. Taguchi K, Fukutomi F, Kuroda A, Kato J, Ohtake H. Genetic identification of chemotactic transducers for amino acids in *Pseudomonas aeruginosa.* Microbiology. 1997;143: 3223-3229.
10. Rico-Jiménez M, Muñoz-Martínez F, García-Fontana C, Fernandez M, Morel M, Ortega A, et al. Paralogous chemoreceptors mediate chemotaxis towards protein amino acids and the non-protein amino acid gamma-aminobutyrate (GABA). Mol Microbiol. 2013;88(6): 1230-1243.
11. McKellar JL, Minnell JJ, Gerth ML. A high-throughput screen for ligand binding reveals the specificities of three amino acid chemoreceptors from Pseudomonas syringae pv. actinidiae. Mol Microbiol. 2015;97(4): 694-707.
12. Brennan CA, DeLoney-Marino CR, Mandel MJ. Chemoreceptor VfcA mediates amino acid chemotaxis in Vibrio fischeri. Appl Environ Microbiol. 2013;79(6): 1889-1896.
13. Nishiyama SI, Suzuki D, Itoh Y, Suzuki K, Tajima H, Hyakutake A, et al. Mlp24 (McpX) of Vibrio cholerae implicated in pathogenicity functions as a chemoreceptor for multiple amino acids. Infect Immun. 2012;80(9): 3170-3178.
14. Pokkuluri PR, Dwulit-Smith J, Duke NE, Wilton R, Mack JC, Bearden J, et al. Analysis of periplasmic sensor domains from Anaeromyxobacter dehalogenans 2CP-C: Structure of one sensor domain from a histidine kinase and another from a chemotaxis protein. Microbiologyopen. 2013;2(5): 766-777.
15. Goers-Sweeney E, Henderson JN, Goers J, Wreden C, Hicks KG, Foster JK, et al. Structure and Proposed Mechanism for the pH-Sensing Helicobacter pylori Chemoreceptor TlpB. Structure. 2012;20(7): 1177-1188.
16. Alvarez-Ortega C, Harwood CS. Identification of a malate chemoreceptor in Pseudomonas aeruginosa by screening for chemotaxis defects in an energy taxis-deficient mutant. Appl Environ Microbiol. 2007;73(23): 7793-7795.
17. Chang C, Tesar C, Gu M, Babnigg G, Joachimiak A, Pokkuluri PR, et al. Extracytoplasmic PAS-like domains are common in signal transduction proteins. J Bacteriol. 2010;192(4): 1156-1159.
18. Kaspar S, Perozzo R, Reinelt S, Meyer M, Pfister K, Scapozza L, et al. The periplasmic domain of the histidine autokinase CitA functions as a highly specific citrate receptor. Mol Microbiol. 1999;33(4): 858-872.
19. Kneuper H, Janausch IG, Vijayan V, Zweckstetter M, Bock V, Griesinger C, et al. The nature of the stimulus and of the fumarate binding site of the fumarate sensor DcuS of Escherichia coli. J Biol Chem. 2005;280(21): 20596-20603.
20. Doan, T. The Bacillus subtilis ywkA gene encodes a malic enzyme and its transcription is activated by the YufL/YufM two-component system in response to malate. Microbiology. 2003;149(9): 2331-2343.
21. Tanaka K, Kobayashi K, Ogasawara N. The Bacillus subtilis YufLM two-component system regulates the expression of the malate transporters MaeN (YufR) and YflS, and is essential for utilization of malate in minimal medium. Microbiology. 2003;149(9): 2317-2329.
22. Reid CJ, Poole PS. Roles of DctA and DctB in signal detection by the dicarboxylic acid transport system of Rhizobium leguminosarum. J Bacteriol. 1998;180(10): 2660-2669.
23. Cheung J, Hendrickson WA. Crystal structures of C4-dicarboxylate ligand complexes with sensor domains of histidine kinases DcuS and DctB. J Biol Chem. 2008;283(44): 30256-30265.
24. Hothorn M, Dabi T, Chory J. Structural basis for cytokinin recognition by Arabidopsis thaliana histidine kinase 4. Nat Chem Biol. 2011;7(11): 766-768.
25. Clarke MB, Hughes DT, Zhu C, Boedeker EC, Sperandio V. The QseC sensor kinase: A bacterial adrenergic receptor. Proc Natl Acad Sci U S A. 2006;103(27): 10420-10425.
26. Cheung J, Bingman CA, Reyngold M, Hendrickson WA, Waldburger CD. Crystal Structure of a Functional Dimer of the PhoQ Sensor Domain. J Biol Chem. 2009;283(20): 13762-12770.
27. Guzman-Verri C, Manterola L, Sola-Landa A, Parra A, Cloeckaert A, Garin J, et al. The two-component system BvrR/BvrS essential for Brucella abortus virulence regulates the expression of outer membrane proteins with counterparts in members of the Rhizobiaceae. Proc Natl Acad Sci U S A. 2002;99(19): 12375-12380.
28. Neiditch MB, Federle MJ, Miller ST, Bassler BL, Hughson FM. Regulation of LuxPQ Receptor Activity by the Quorum-Sensing Signal Autoinducer-2. Mol Cell. 2005;18: 507-518.
29. Zere TR, Vakulskas CA, Leng Y, Pannuri A, Potts AH, Dias R, et al. Genomic Targets and Features of BarA-UvrY (-SirA) Signal Transduction Systems. PLoS One. 2015;10(12): e0145035.
30. Camacho MI, Alvarez AF, Chavez RG, Romeo T, Merino E, Georgellis D. Effects of the Global Regulator CsrA on the BarA/UvrY Two-Component Signaling System. J Bacteriol. 2015;197(5): 983-991.
31. Cox AJ, Hunt ML, Ruffolo CG, Adler B. Cloning and characterisation of the *Pasteurella multocida ahpA* gene responsible for a haemolytic phenotype in *Escherichia coli*. Vet Microbiol. 2000;72: 135-152.
32. Monterrubio R, Baldoma L, Obradors N, Aguilar J, Badia J. A Common Regulator for the Operons Encoding the Enzymes Involved in D-Galactarate, D-Glucarate, and D-Glycerate Utilization in Escherichia coli. J Bacteriol. 2000;182(9): 2672-2674.
33. Minasov G, Padavattan S, Shuvalova L, Brunzelle JS, Miller DJ, Baslé A, Massa C, Collart FR, Schirmer T, Anderson WF. Crystal structures of YkuI and its complex with second messenger cyclic Di-GMP suggest catalytic mechanism of phosphodiester bond cleavage by EAL domains. J Biol Chem. 2009;284(19): 13174-13184.
