# Supplementary material for: Cache Domains That are Homologous to, but Different from PAS Domains Comprise the Largest Superfamily of Extracellular Sensors in Prokaryotes
Source: PLoS Comput Biol. 2016 Apr 6;12(4):e1004862. doi: 10.1371/journal.pcbi.1004862 (PMC4822843; doi:10.1371/journal.pcbi.1004862)
Supplement: S9 Table — (DOCX) [file pcbi.1004862.s015.docx]

**S9 Table. Cellular localization prediction for members of the Cache superfamily using TMHMM.**

| **Family** | **Total** | **Between 2 TM or TM and HAMP**  **(Extracellular)** | **No TM**  **(Intracellular)** |
| --- | --- | --- | --- |
| dCache_1 | 15569 | 12252 | 1004 |
| dCache_2 | 299 | 249 | 14 |
| dCache_3 | 883 | 787 | 11 |
| Cache_3-Cache_2 | 407 | 351 | 11 |
| sCache_2 | 2243 | 1783 | 174 |
| sCache_3_1 | 2854 | 2658 | 9 |
| sCache_3_2 | 2499 | 2253 | 17 |
| sCache_3_3 | 276 | 249 | 3 |
| Diacid_rec | 1274 | 0 | 1268 |
| CHASE | 1214 | 861 | 58 |
| 2CSK_N | 966 | 834 | 19 |
| SMP_2 | 788 | 439 | 31 |
| DUF2222 | 713 | 695 | 1 |
| PhoQ_Sensor | 556 | 542 | 0 |
| CHASE4 | 529 | 411 | 35 |
| Stimulus_sens_1 | 203 | 201 | 1 |
| YkuI_C | 184 | 0 | 180 |
| LuxQ-periplasm | 115 | 84 | 2 |
| **Cache clan** | **31572** | **24649 (78.07 %)** | **2838 (8.99%)** |
| **PAS clan** | **88093** | **573 (0.65%)** | **65496 (74.34%)** |
