# Supplementary material for: Cache Domains That are Homologous to, but Different from PAS Domains Comprise the Largest Superfamily of Extracellular Sensors in Prokaryotes
Source: PLoS Comput Biol. 2016 Apr 6;12(4):e1004862. doi: 10.1371/journal.pcbi.1004862 (PMC4822843; doi:10.1371/journal.pcbi.1004862)
Supplement: S10 Table — Domain models were searched against non-redundant prokaryotic extracellular sequences (DOCX) [file pcbi.1004862.s016.docx]

**S10 Table. Abundance of the two largest clans in known prokaryotic extracellular sensory domains.** Domain models were searched against non-redundant prokaryotic extracellular sequences.

| **Clan** | **Family** | **Count** |
| --- | --- | --- |
| Cache | dCache_1 | 9570 |
|  | sCache_3_1 | 1481 |
|  | sCache_2 | 1347 |
|  | sCache_3_2 | 1221 |
|  | CHASE | 690 |
|  | dCache_3 | 678 |
|  | 2CSK_N | 670 |
|  | CHASE4 | 365 |
|  | Cache_3-Cache_2 | 302 |
|  | DUF2222 | 294 |
|  | dCache_2 | 226 |
|  | sCache_3_3 | 208 |
|  | Sensor_TM1 | 153 |
|  | Stimulus_sens_1 | 149 |
|  | PhoQ_Sensor | 133 |
|  | LuxQ-periplasm | 63 |
|  | **Total** | **17550** |
| 4HB_MCP | 4HB_MCP_1 | 2484 |
|  | TarH | 1164 |
|  | CHASE3 | 1034 |
|  | **Total** | **4682** |
