# Supplementary material for: Cache Domains That are Homologous to, but Different from PAS Domains Comprise the Largest Superfamily of Extracellular Sensors in Prokaryotes
Source: PLoS Comput Biol. 2016 Apr 6;12(4):e1004862. doi: 10.1371/journal.pcbi.1004862 (PMC4822843; doi:10.1371/journal.pcbi.1004862)

dCache_1

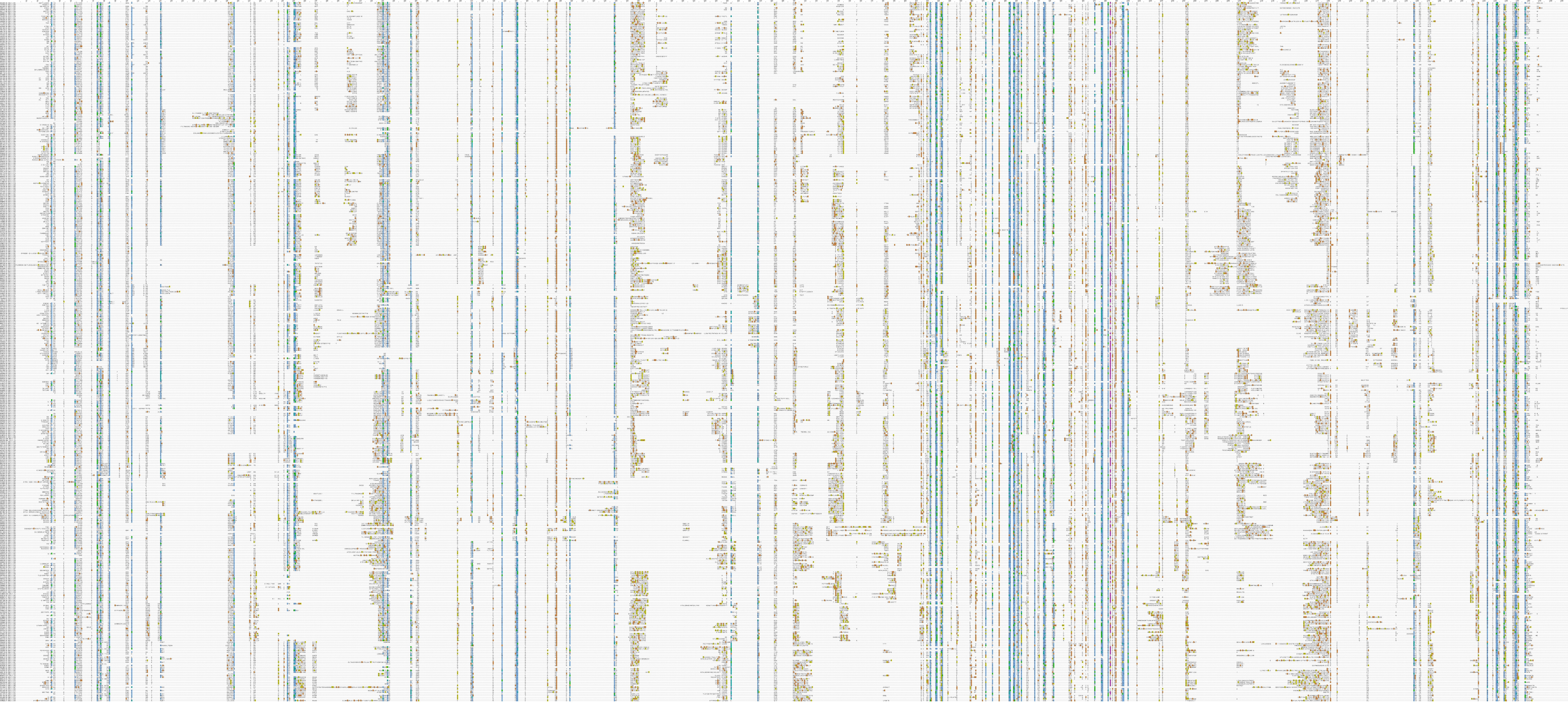

dCache_2

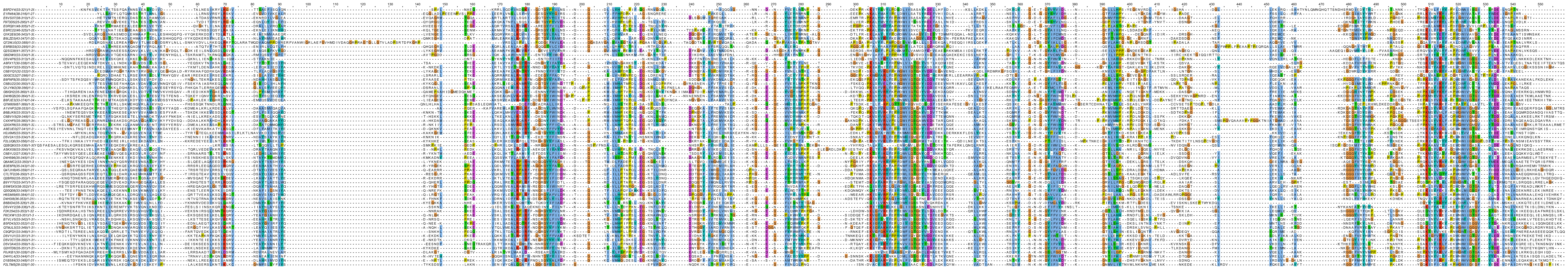

dCache_3

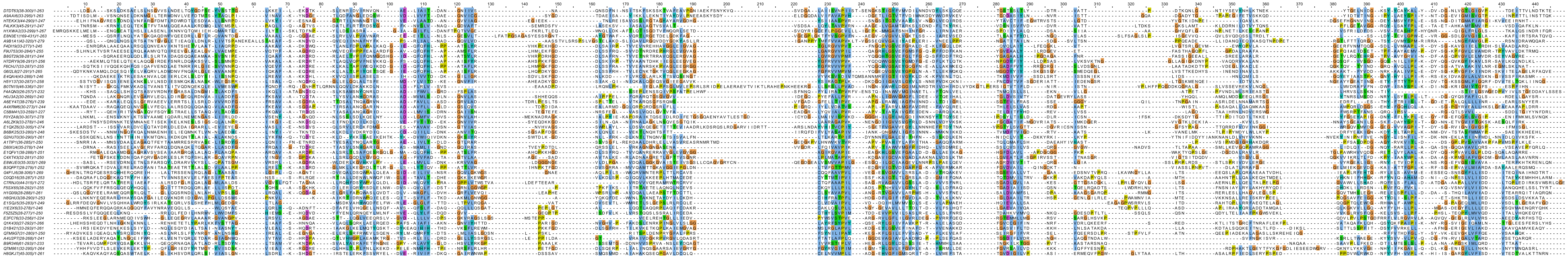

Cache_3-Cache_2

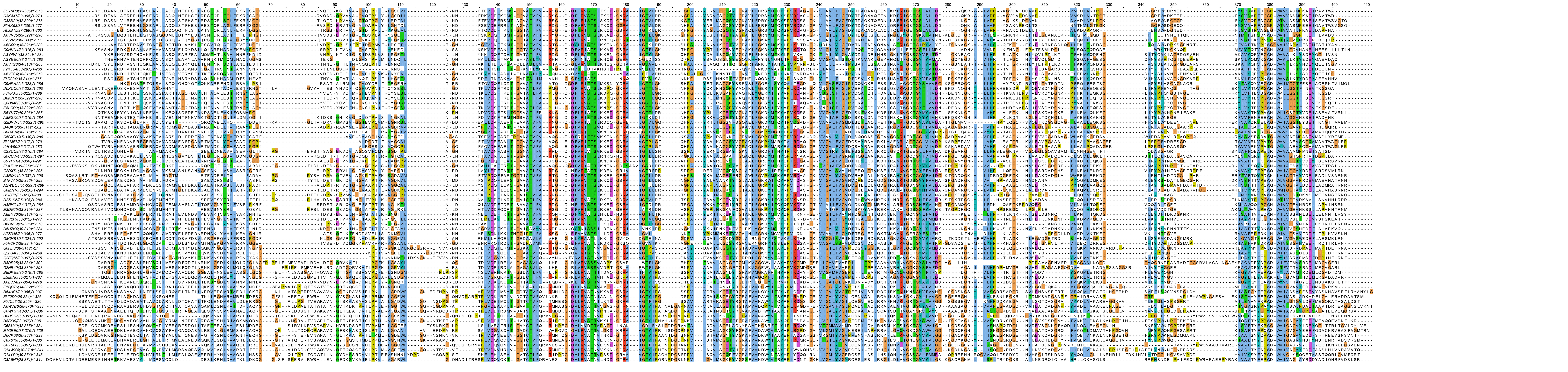

sCache_2

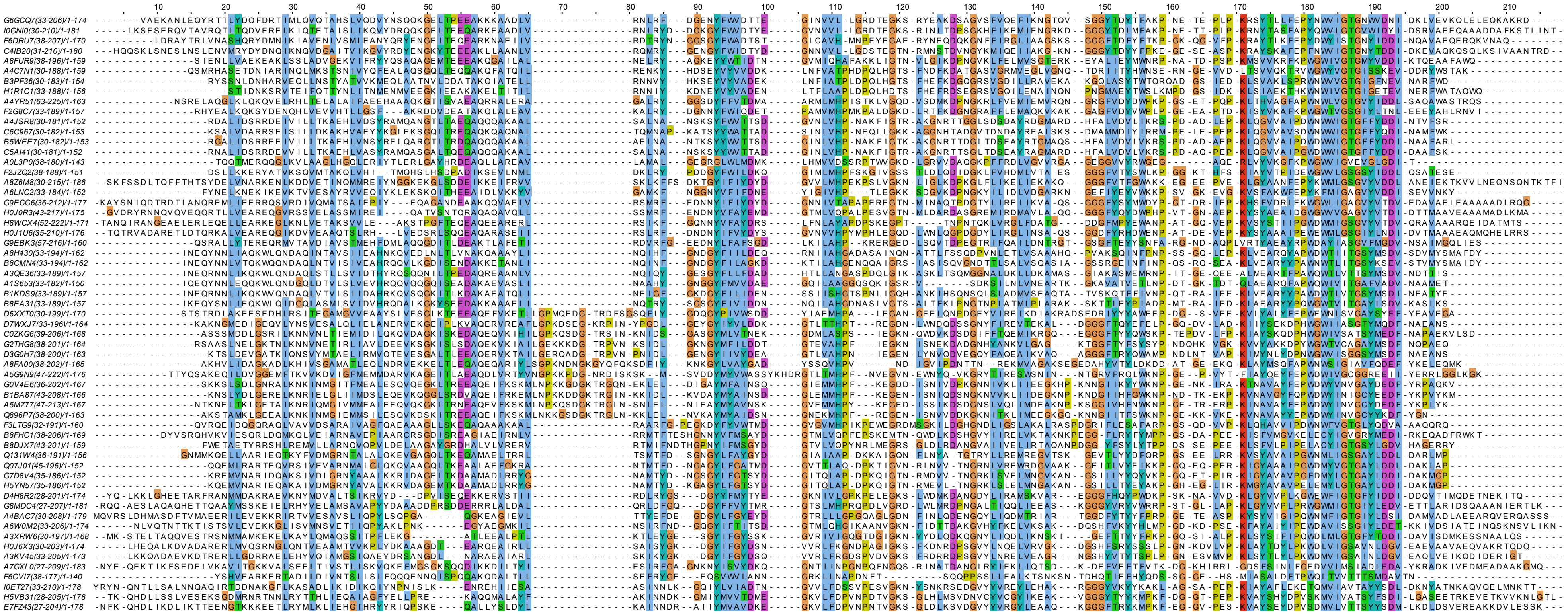

sCache_3_1

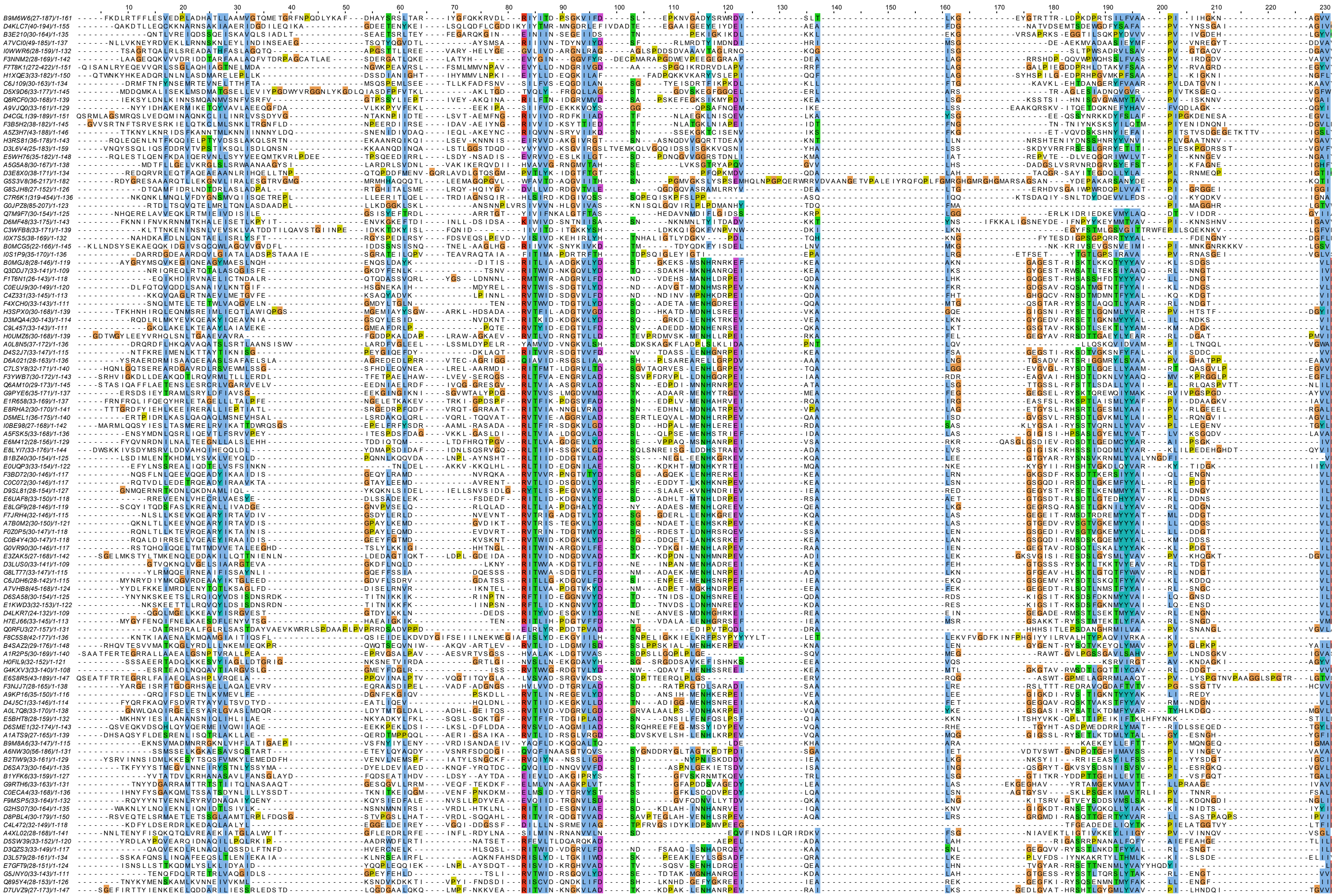

sCache_3_2

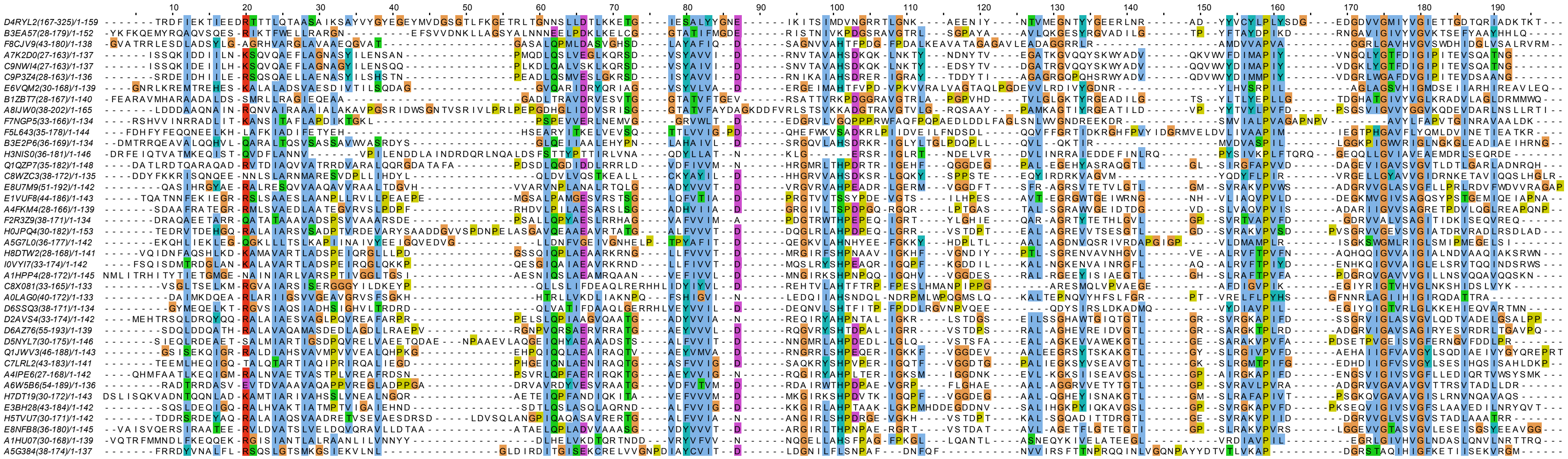

sCache_3_2

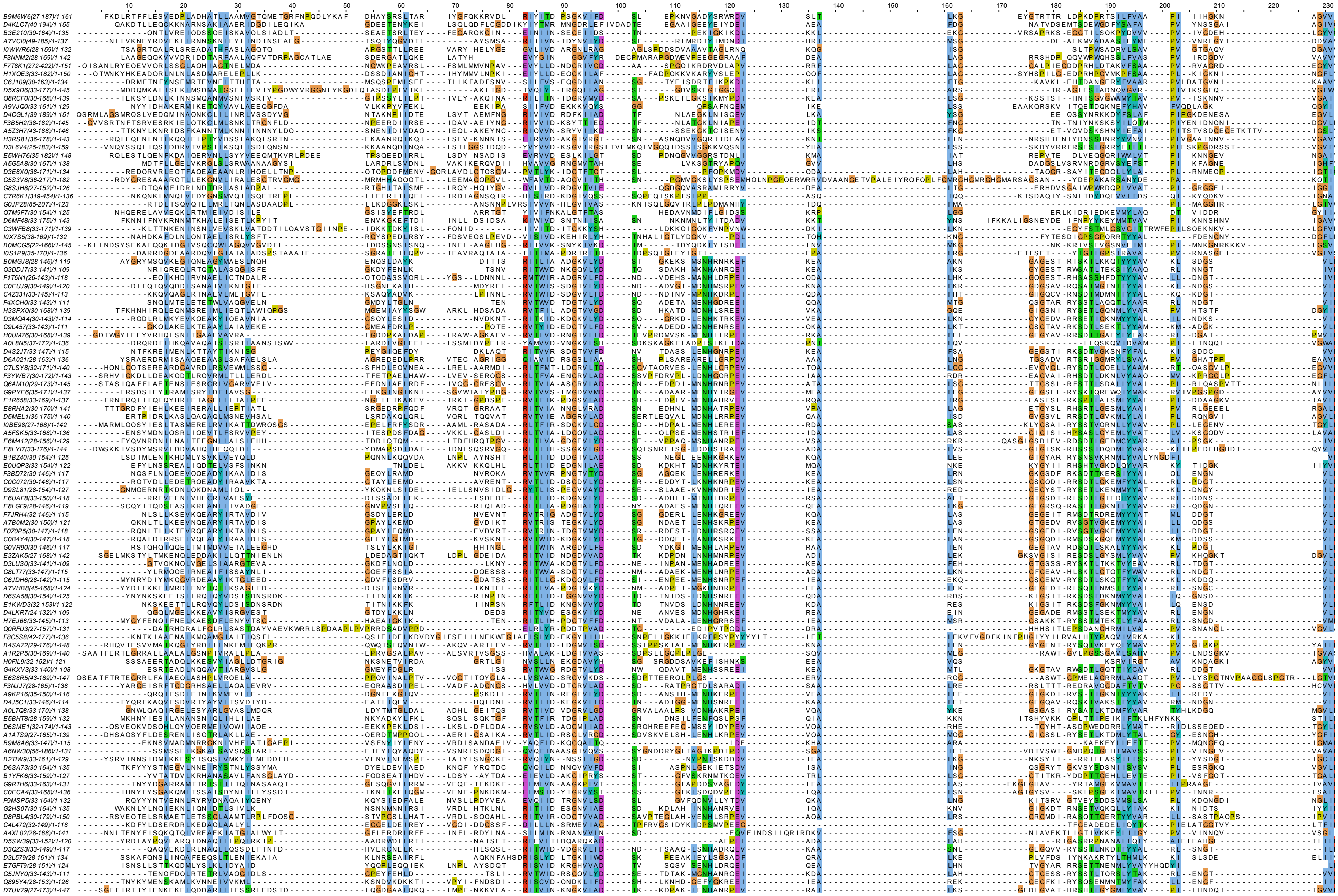

Supplement: S1 Data — (PDF) [file pcbi.1004862.s017.pdf]
